# Supplementary material for: Nonlinear machine learning pattern recognition and bacteria-metabolite multilayer network analysis of perturbed gastric microbiome
Source: Nat Commun. 2021 Mar 26;12:1926. doi: 10.1038/s41467-021-22135-x (PMC7997970; doi:10.1038/s41467-021-22135-x)
Supplement: Supplementary file 1 — Supplementary Information [file 41467_2021_22135_MOESM1_ESM.pdf]

# Additional file 1: Supplementary notes, figures and tables

## Table of contents

**Supplementary Note 1:** MCE to unsupervisedly infer and visualize phylogenetic (hierarchical) relations.

**Supplementary Note 2:** Relative performance improvement.

**Supplementary Note 3:** Nonlinear dimension reduction techniques t-SNE and Isomap.

**Supplementary Note 4:** Artificial Datasets

**Supplementary Note 5:** Dimensionality Reduction analysis in artificial datasets

**Supplementary Note 6:** Origin of the Paroni Sterbini data nonlinearity

**Supplementary Note 7:** Normalizations applied in microbiome studies

**Supplementary Note 8:** From nonlinear data to linear analysis

**Supplementary Note 9:** Computing platforms adopted to implement the algorithms.

**Supplementary Figure 1:** The Tripartite-Swiss-Roll as an example of data nonlinear organization.

**Supplementary Figure 2:** Nonlinear dimension reduction by t-SNE and Isomap applied to the Paroni Sterbini dataset.

**Supplementary Table 1:** Results of unsupervised analysis on the 'microbial-like' synthetic dataset.

**Supplementary Figure 3:** LDA analysis of gastric biopsies dataset (Paroni Sterbini *et al.*).

**Supplementary Figure 4:** Pairwise PCA of Paroni Sterbini's gastric samples.

**Supplementary Figure 5:** Pairwise PCA of the Tripartite-Swiss-Roll.

**Supplementary Figure 6:** MCE on gastric biopsies dataset (Paroni Sterbini *et al.*), restricted to PPI-treated patients.

**Supplementary Table 2:** Average PSI-ROC and PSI-PR best results and trustworthiness with standard error on the real datasets, when applying Leave-one-out-cross-validation (LOOCV).

**Supplementary Table 3:** PSI-ROC and PSI-PR results on the datasets after approximation to the negative binomial distribution.

**Supplementary Table 4:** Rank performance on the datasets after approximation to the negative binomial distribution.

**Supplementary Table 5:** Clustering results on the datasets after approximation to the negative binomial distribution.

**Supplementary Table 6:** PSI-ROC and PSI-PR results on the rarefied datasets.

**Supplementary Table 7:** Rank performance on the rarefied datasets.

**Supplementary Table 8:** Clustering results on the rarefied datasets.

**Supplementary Figure 7:** PCA analysis reveals separation related to PPI-treatment in gastric fluid.

**Supplementary Figure 8:** PC-corr network to investigate the effect of PPI treatment on gastric fluid.

**Supplementary Figure 9:** PCA analysis reveals separation related to PPI-treatment in gastric mucosa, in the patients negative to *H. pylori* test.

**Supplementary Figure 10:** PC-corr network to investigate the effect of PPI treatment on gastric mucosa.

**Supplementary Figure 11**: The overlap between Amir *et al.* and Paroni Sterbini *et al.* networks, related to PPI treatment in dyspepsia, is statistically significant and hence it cannot be generated by a random process.

**Supplementary Figure 12:** In Paroni Sterbini *et al.* dataset, PCA analysis reveals separation related to *H. pylori* infection in gastric tissue, in the PPI-untreated patients.

**Supplementary Figure 13:** PC-corr network to investigate the effect of *H. pylori* infection on gastric mucosal microbiota in Paroni Sterbini *et al.* data.

**Supplementary Figure 14:** In Parsons *et al. dataset*, PCA analysis can significantly discriminate gastric mucosal biopsy specimens according to *H. pylori*-positivity.

**Supplementary Figure 15:** PC-corr network to investigate the effect of *H. pylori* infection on gastric mucosal microbiota in Parsons *et al.* data.

**Supplementary Figure 16:** The overlap between Paroni Sterbini *et al.* and Parsons *et al.* networks, exemplifying the effect of *H. pylori* infection on gastric mucosal microbiota, is statistically different from a random overlap.

**Supplementary Figure 17:** Full PPI-affected bacteria-metabolite network in gastric environment of dyspeptic patients.

**Supplementary Figure 18:** Full *H. Pylori*-affected bacteria-metabolite network in gastric environment of dyspeptic patients.

**Supplementary Figure 19:** Example of trustworthiness computation.

**Supplementary Table 9:** List of primers for each dataset.

**Supplementary Data 1:** Excel file with class segregation p-value significance at different embedding dimensions.

**Supplementary Data 2:** Excel file with PSI performances on 16S rRNA gene amplicons data.

**Supplementary Data 3:** Excel file with Paroni Sterbini data.

**Supplementary Data 4:** Excel file with LDA for dimension reduction.

**Supplementary Data 5:** Excel file with clustering results on real data.

**Supplementary Data 6:** CSV file with PPI-affected bacteria-metabolite network pathway enrichment analysis.

**Supplementary Data 7:** CSV file with *H. pylori*-affected bacteria-metabolite network pathway enrichment analysis.

**Supplementary Data 8:** Excel file with full results of unsupervised analysis on the 'microbial-like' synthetic dataset.

**Supplementary Data 9:** Excel file with full PSI-ROC and PSI-PR results on the datasets after approximation to the negative binomial distribution.

**Supplementary Data 10:** Excel file with full clustering results on the datasets after approximation to the negative binomial distribution.

**Supplementary Data 11:** Excel file with full PSI-ROC and PSI-PR results on the rarefied datasets.

**Supplementary Data 12:** Excel file with full clustering results on the rarefied datasets.

**Supplementary Data 13:** Excel file with 'microbial-like' dataset.

**Supplementary Data 14:** Excel file with Tripartite-Swiss-Roll dataset.

# Supplementary Note 1

## *MCE to unsupervisedly infer and visualize phylogenetic (hierarchical) relations*

A previous study by Alanis-Lobato *et al.* [1] showed that MCE is automatically able to unsupervisedly infer and visualize phylogenetic (hierarchical) relations directly from individual SNP profiles in human population genetics. Precisely, ncMCE detected separation between ethnic groups and provided an ordering over the discriminating dimension that was related to the phylogenetic organization of these populations.

This ability of MCE to infer and visualize phylogenetic (hierarchical) relationships was confirmed in our study on the Paroni Sterbini *et al.* dataset [2] (see Results section-'Gastric tissue dataset unsupervised analysis'). As mentioned in the main text (see section 'PCA, MDS (or PCoA) and LDA'), MDSwUF uses a weighted Unifrac distance that combines the prior knowledge of the bacterial phylogenetic tree with the information given by the bacterial abundance. Here we show that MCE perform better than MDSwUF on the Paroni Sterbini *et al.* dataset, due to its ability to infer the (hierarchical) phylogenetic relationship among the bacteria directly from the bacterial abundance of the dataset, by performing a hierarchical embedding. Hence, MCE can be used to compare the composition of microbial communities in the studied samples, where the phylogenetic information is instead directly inferred from bacterial abundance, differently from MDSwUF.

# Supplementary Note 2

## *Relative performance improvement*

The performance improvement becomes evident when "quantifying the improvement in terms of the distance from the exact predictor". As a didactic example, let us compare the current PSI-ROC

improvement of 0.06 (0.85 – 0.91) against a case with a same hypothetical improvement but closer to randomness (0.50 – 0.56). In the former the relative improvement in respect to the exact predictor is 40% (computed as (0.91-0.85)/(1-0.85)*100), whereas in the latter is 12% (computed as (0.56-0.50)/(1-0.50)*100). Similarly for PSI-PR, MCE (PSI-PR=0.96) relative improvement from PCA (PSI-PR=0.91) in respect to the perfect predictor is 56% (computed as (0.96-0.91)/(1-0.91)*100).

# Supplementary Note 3

## *Nonlinear dimension reduction techniques t-SNE and Isomap*

As we stressed in the introduction, t-SNE and Isomap algorithms require optimal tuning of parameters (two for t-SNE and one for Isomap). We believe that advanced nonlinear data analysis needs adaptiveness and automatization, whereas methods such as t-SNE and Isomap, although in principle are unsupervised, in practice are applied in a supervised manner and the hypothesized class labels are used to learn their best parameter tuning. Unlikely, in small size datasets, parameter tuning is a relevant issue that may cause overfitting, especially with more than one parameter such as in the case of t-SNE and, to the best of our knowledge, there is not yet any commonly accepted solution for this. Here, with the mere intention to provide a proof of concept that allows to compare MCE with other nonlinear dimension reduction methods, we apply a supervised procedure in which the labels are used to supervisedly tune the internal parameters of these methods and we select the solution which offers the best performance as seen in Supplementary Figure 2. t-SNE (PSI-ROC: 0.90, PSI-PR: 0.94) and Isomap (PSI-ROC: 0.87, PSI-PR: 0.94) display difficulty to resolve the difference between treated and untreated samples, mostly for the cases of treated patients (blue points) and untreated patients without *H. Pylori* infection (red points). This indicates

that in principle adaptive parameter-free algorithms such as MCE may also outperform more complex algorithms under difficult scenarios such as for this particular case.

# Supplementary Note 4

## *Artificial datasets*

In order to test and visualize how the algorithms could detect nonlinearity, we performed the analyses on two artificial datasets: (1) The Tripartite-Swiss-Roll dataset: an artificial dataset characterized by nonlinear structures and generated as discretization of the manifold associated to a Swiss-Roll function [3] in a three-dimensional (3D) space. Indeed, it is a synthetic dataset obtained as the partition in three sections of a discrete Swiss-Roll manifold depicted in a three-dimensional space [3]. It reproduces the typical nonlinearity (given by the Swiss-Roll shape) and the discontinuity (given by the tripartition of the manifold), that we do not see and that are often hidden in the multidimensional representation of our samples. See the illustration in the original 3D-space of the Tripartite-Swiss-Roll dataset in Supplementary Figure 1a. This dataset is useful to introduce readers, not expert with nonlinear data analysis, to the basic concepts of nonlinear dimension reduction and therefore to facilitate their understanding of the new proposed methodologies for nonlinear dimension reduction. And (2) A Microbial-like dataset: a synthetic dataset generated with the R function rmvzinegbin from the library SpiecEasi [4] that simulates a microbial OTU table. The dataset contains four different groups with 50 samples each and 200 features. Two type of noise were introduced with the aim to recreate non-linearity, strategy already applied - with a different purpose - in the study by Lo & Marculescu [5]. The first type of noise introduces 0 elements to counts that are non-zero, whilst the second type introduces a deviation/noise from the true non-zero count.

# Supplementary Note 5

## *Dimensionality Reduction analysis in artificial datasets*

The Tripartite-Swiss-roll dataset (that is a synthetic dataset containing nonlinear structures obtained by tri-partitioning a discrete Swiss-Roll manifold [3] in a three-dimensional space), presents a hierarchical-organized nonlinearity (Supplementary Figure 1a). In this case, similarly to the result of the Paroni Sterbini *et al.* analysis, non-centred MCE is able to perform a hierarchical embedding that orders the hidden subgroups of the dataset along the second dimension of embedding (Supplementary Figure 1e). On the contrary, PCA, MDSbc and NMDS (Supplementary Figure 1b-d) were unable to resolve the nonlinearity of the Tripartite-Swiss-Roll: its three partitions are either superimposed (Supplementary Figure 1b, d) or twisted in a horseshoe shape (Supplementary Figure 1c). Indeed, the Tripartite-Swiss-Roll is purposely created to reproduce a manifold that is nonlinear and discontinuous (broken in three parts) such as the results of MCE analysis of Paroni Sterbini *et al.* seems to be. Furthermore, to compare the different approaches in a more "realistic" scenario, a synthetic microbial-like dataset (which resamples the nonlinearities encountered in the Paroni Sterbini *et al.* data) is generated and analysed. MCE overcomes once again the other dimensionality reduction techniques and is very close to guarantee a separability equivalent to the one obtained in the high dimensional space (HD) (Supplementary Table 1). These results are similar to the ones obtained in the real datasets. As expected, MDS with weighted Unifrac distance is highly affected by the fact that phylogenetic information between synthetic features is not available and it is directly extracted from the OTU table. Interestingly, and opposite to what already shown in the real dataset, MDS with Theta-YC distance obtains great performances close to MCE.

# Supplementary Note 6

## *Origin of the Paroni Sterbini data nonlinearity*

Moreover, because of the discovered major nonlinear complexity in the Paroni Sterbini gastric biopsy dataset, we wanted to verify whether it was generated by multi-grouping (three-body interaction problem associated to the presence of three hidden clusters). To do so, we applied PCA to three subsampled versions of the dataset (with the best normalization originally found for the complete dataset), each corresponding to the combination of two groups (Supplementary Figure 4), and PCA could find significant separation (PSI-ROC and PSI-PR > 0.80). To further confirm that the presence of multiple sample groups generates the data complexity, we did the same for the Tripartite Swiss-Roll (Supplementary Figure 5), where we recovered the discrimination, even though two comparisons overlap to some extent (Supplementary Figure 5a and c). Additionally, it might be argued that the presence of *H. pylori* only drives the difference of the microbial community, instead of PPI treatment. However, if this were the case, then the segregation between H+ and H- samples would be evident as well inside the PPI treated group. However, the p-values from the two-sided Mann-Whitney test are not anymore significant for this case (p-value PCA: 0.46 & p-value MCE: 1) and no evident segregation arises neither by eyes, as supported by the Supplementary Figure 6.

# Supplementary Note 7

## *Normalizations applied in microbiome studies*

We have to clarify that normalizations besides scaling (DRS and DCS) and log-transformation (log(1+$x$)) could potentially lead to different performance results of unsupervised analysis. Normalization is crucial to address uneven sampling depth and sparsity (high proportion of zeros)

in microbiome data, like rarefying an OTU table, that is randomly sampling without replacement from each sample such that all samples have the same number of total counts (sequencing depth) [6–9] (http://qiime.org/scripts/single_rarefaction.html). This normalization is recommended to moderate the sensitivity of UniFrac distances to sequencing (sampling) depth [10,11], especially differences in the presence of rare OTUs [12], nonetheless it is also considered statistically improper due to the omission of data [10].

Another normalization was introduced in 2010 by Anders and colleagues for general sequence count data (function *varianceStabilizingTransformation* implemented in the Bioconductor DESeq2 package), that uses a Variance-Stabilization Transformation (VST) by modelling microbiome count data with Negative Binomial (NB) distribution [7,10].

We also provide the results with these two different normalizations, and we further confirm that the data are segregated in the HD space when pre-processed according to them, as shown in the PSI-ROC and PSI-PR tables in Additional file (for negative binomial, Supplementary Tables 3 and 4 and Supplementary Data 9; for rarefaction, Supplementary Tables 6 and 7 and Supplementary Data 11). Interestingly, across all the datasets MCE decreases its performance with these pre-processing techniques, and remarkably with rarefied datasets. Instead, other linear techniques improve in performance (Supplementary Table 3 for negative binomial; Supplementary Table 6 for rarefaction), suggesting that these adjustments linearize the datasets. Indeed, since MCE is a hierarchical technique, it needs the presence of nonlinearity to perform well. In a similar way, with these two normalizations the accuracy of MC-MCL drops down (less remarkably in the rarefaction datasets), while the performance of MCL does not increment (Supplementary Table 5 and Supplementary Data 10 for negative binomial; Supplementary Table 8 and Supplementary Data 12 for rarefaction). It is true that some pre-processing steps such as negative binomial tend

to linearize the data but, in this manner, they can also remove important nonlinear discriminative information, as we show with the results of unsupervised analysis. Therefore, some pre-processing approaches can also cancel important nonlinear discriminant information present in the analysed data.

# Supplementary Note 8

## *From nonlinear data to linear analysis*

Up to this point, in order to assess the emergence of nonlinear patterns in data, the application and performance of linear and non-linear dimensionality reduction algorithms has been compared. Special focus was on Paroni Sterbini dataset, where the presence or absence of *H. pylori* infection in addition to the medical treatment (or not) with PPI medicaments created a complex nonlinear scenario difficult to disentangle using linear transformations and even some nonlinear ones. Then, with the didactic help of the Tripartite-Swiss-roll dataset, we clarified that the origin of the Paroni Sterbini nonlinearity stays in the three-body problem. Indeed, considering pairwise comparisons of only two groups, the nonlinearity vanished. Based on these considerations, now we conduct only the two-group comparison of PPI treated/nontreated patients in which presence of *H. Pylori* was negative, since these data are available both in Paroni Sterbini and Amir. Such simplification of the investigation enables the application of the mentioned PC-corr algorithm, since, for the binary class problem both Paroni Sterbini and Amir4 datasets present a significant segregation measured by Mann-Whitney p-value when embedded by the linear algorithm PCA.

# Supplementary Note 9

## *Computing platforms adopted to implement the algorithms*

Dimensionality reduction was performed in MATLAB on the abundance matrix of genus-level taxonomic assignments, with samples in rows and taxonomic assignments (OTUs) in columns. For MDSwUF, the computation of the weighted UniFrac distance was performed in R. We used the following MATLAB functions to calculate PCA, MDS and NMDS (Sammon Mapping) respectively: *svd*, *cmdscale* and *mdscale*. For the calculation of the Theta YC distance, the mothur [13] approach was implemented in MATLAB. For the calculation of Bray-Curtis dissimilarity, we used the function MATLAB *f_braycurtis* in the Fathom Toolbox [14] (http://www.marine.usf.edu/user/djones/matlab/matlab.html). Instead, for the calculation of the weighted Unifrac distance for all sample pairs, we used the R function *UniFrac* in the phyloseq package (https://bioconductor.org/packages/release/bioc/html/phyloseq.html), after creating a phyloseq-class object (with R function *phyloseq* in the same package) that contains both the abundance table (OTU table) and the phylogenetic tree. The MATLAB code for MCE/ncMCE is available online at: https://sites.google.com/site/carlovittoriocannistraci/5-datasets-and-matlab-code/minimum-curvilinearity-ii-april-2012. For MCL clustering, we installed the MCL-edge software (http://micans.org/mcl/) in a Windows environment, following the procedure suggested by the authors in the software website. To apply this algorithm, we created a MATLAB function that generates automatically the input for MCL (equivalent to the mcxarray function in the software) and then uses a system call to run MCL in a UNIX-like environment (Cygwin, https://www.cygwin.com/). PC-corr method was performed in MATLAB on the abundance matrix of the genus-level taxonomic assignments, with samples in rows and taxonomic assignments in columns. The PC-corr algorithm is available as MATLAB function (as well as R function) at:

https://github.com/biomedical-cybernetics/PC-corr_net. Then the obtained PC-corr and bacteria-metabolite networks were displayed by Cytoscape (http://www.cytoscape.org/).

## Supplementary Figures and Tables

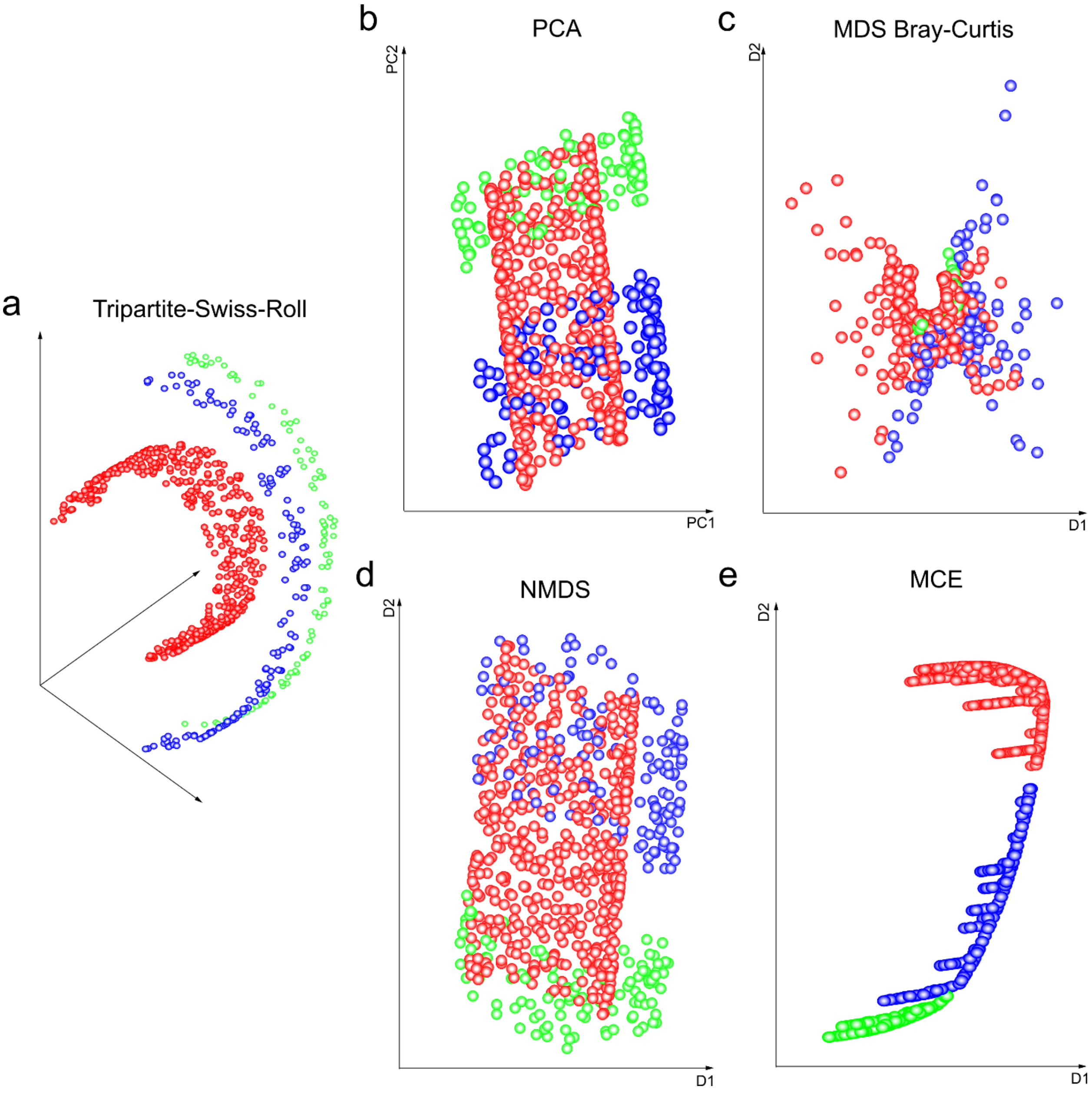


**Supplementary Figure 1. The Tripartite-Swiss-Roll as an example of data nonlinear organization.**

a) Tripartite-Swiss-Roll; b) PCA; c) MDS (Bray-Curtis dissimilarity); d) NMDS (Sammon Mapping); e) MCE. The three different colours (red, blue and green) represent the three partitions of the Swiss-roll

manifold. This figure shows the inability of PCA, MDS and NMDS to reveal the inner nonlinear structure of the Tripartite-Swiss-Roll, which appears collapsed (b, d) or with a horseshoe shape (c) in comparison with an approach tailored for elucidate nonlinear structures MCE (e).

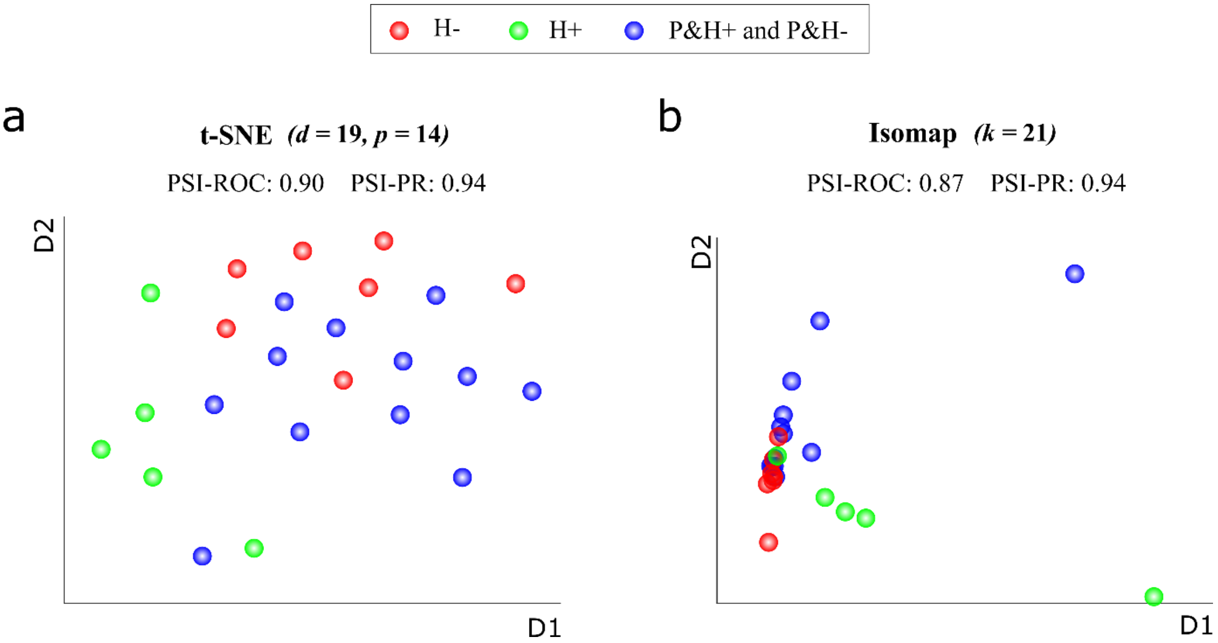


**Supplementary Figure 2. Nonlinear dimension reduction by t-SNE and Isomap applied to the Paroni Sterbini dataset.** The plots report the best t-SNE and Isomap results based on PSI-ROC and PSI-PR for the separation of the three different groups (PPI-treated [blue points], untreated H+ [green points] and untreated H- [red points]), evaluated in the 2D embedding space. a) Best t-SNE embedding obtained with parameters dimension 19 and perplexity 14; b) Best Isomap embedding obtained with parameter *k* nearest neighbors of 21. Note that the sample labels were used in order to supervised select the best input parameters for each algorithm.

**Supplementary Table 1. Results of unsupervised analysis on the ‘microbial-like’ synthetic dataset.** Best results of unsupervised dimension reduction techniques according to PSI-ROC and PSI-PR, which are indices for evaluation of sample separation in the space of the first two dimensions of embedding. HD (high dimension) indicates the separability in the high dimensional space (no dimension reduction) and it represents a reference to compare with the separability after dimension reduction. Results are ordered from the best (top) to the worst (bottom) performance. For each PSI value, the respective trustworthiness is reported.

| Method | PSI-ROC | Trust | PSI-PR | Trust |
|---|---|---|---|---|
| **HD** | 1.00 | 0.0009 | 1.00 | 0.0009 |
| **MCE** | 0.99 | 0.0009 | 0.99 | 0.0009 |
| **MDStyc** | 0.97 | 0.0009 | 0.97 | 0.0009 |
| **MDSbc** | 0.96 | 0.0009 | 0.96 | 0.0009 |
| **PCA** | 0.85 | 0.0009 | 0.85 | 0.0009 |
| **NMDS** | 0.85 | 0.0009 | 0.85 | 0.0009 |
| **MDSwUF** | 0.83 | 0.0023 | 0.83 | 0.0020 |

Note: all PSI-ROC and PSI-PR values can be found in Supplementary Data 8

**Abbreviations:** HD: High Dimension; MCE: Minimum Curvilinear Embedding; MDSbc: Multidimensional Scaling with Bray-Curtis dissimilarity; MDSwUF: Multidimensional Scaling with weighted UniFrac distance; NMDS: Non-metric Multidimensional Scaling; MDStyc: Multidimensional Scaling with Theta-YC distance; PCA: Principal Component Analysis; PSI-ROC: Projection Separability Index measured by Area Under the Curve; PSI-PR: Projection Separability Index measured by Area Under the Precision Recall; Trust: Trustworthiness.

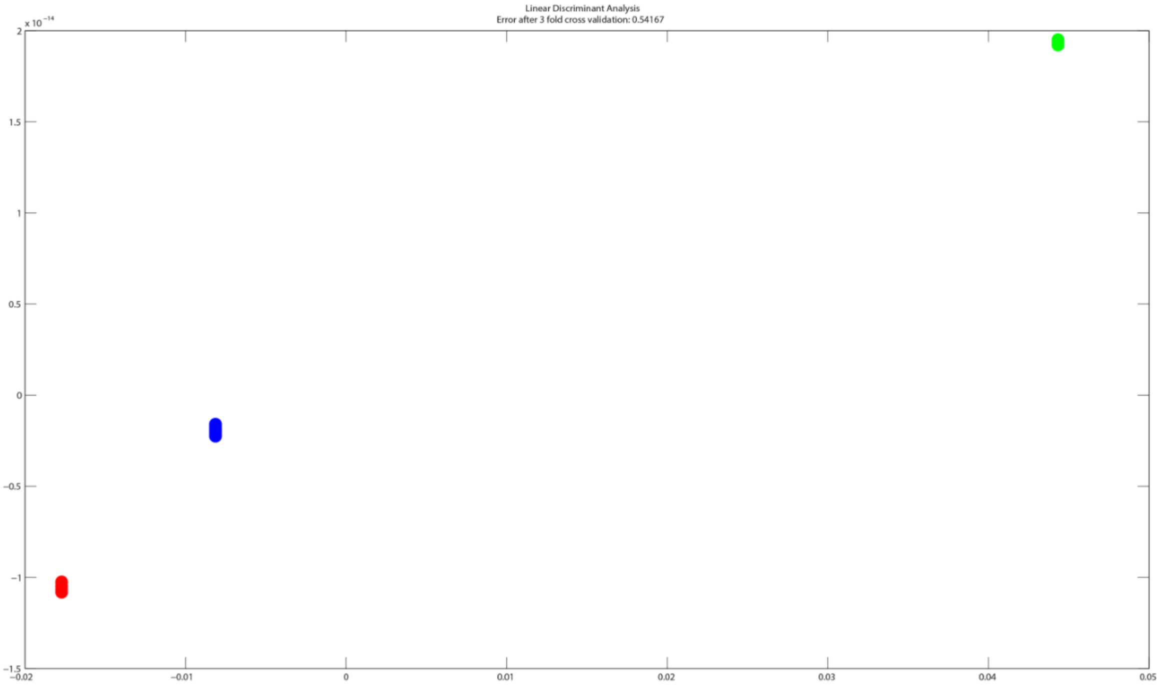


**Supplementary Figure 3. LDA analysis of gastric biopsies dataset (Paroni Sterbini *et al.*).** The cross-validation test showed that this constrained technique could re-assign samples to their three groups with 54% of error, confirming its statistical invalidity for the small size dataset problem.

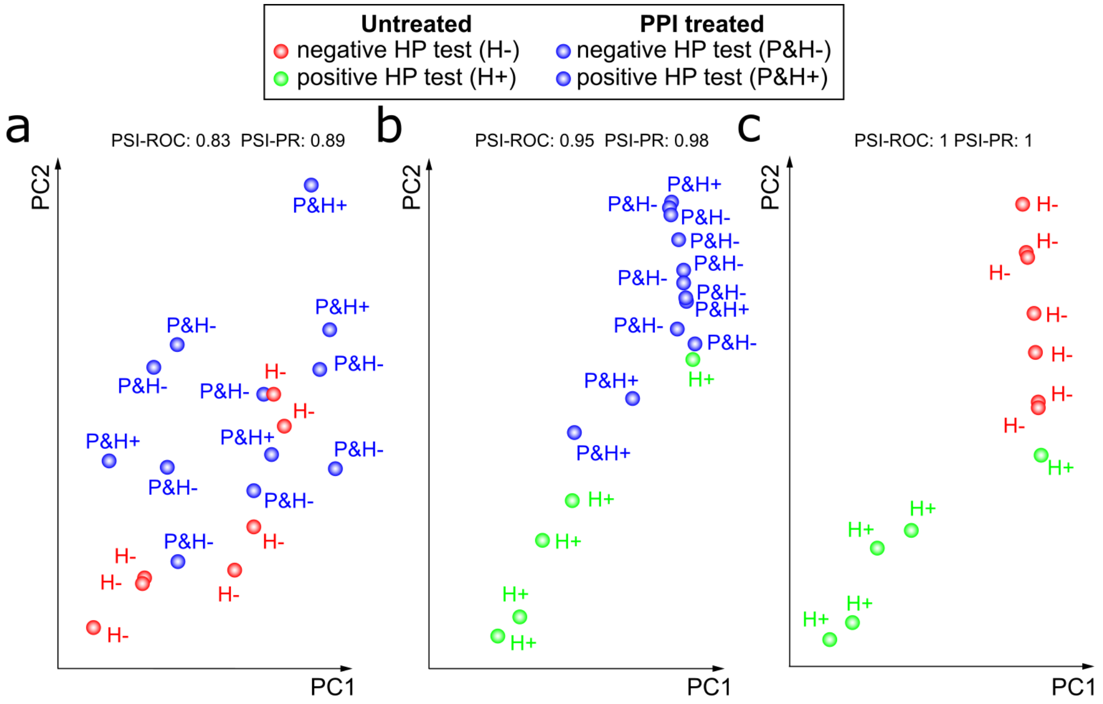

**Supplementary Figure 4. Pairwise PCA of Paroni Sterbini's gastric samples.** PCA was applied to three subsampled versions of the Paroni Sterbini dataset (keeping the best normalization found for the original dataset), each corresponding to the combination of two groups: (a) PPI-treated and untreated H. pylori negative samples; (b) PPI-treated and untreated H. pylori positive samples; (c) untreated H. pylori negative and untreated H. pylori positive samples. The PSI-ROC and PSI-PR are reported as well as overall estimators of separation between the groups in the 2D reduced space.

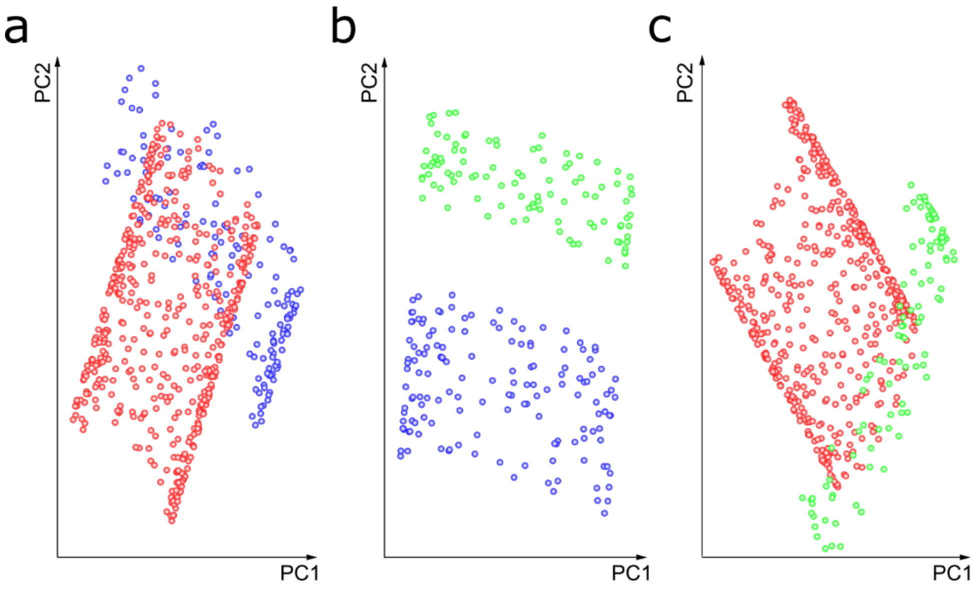


**Supplementary Figure 5. Pairwise PCA of the Tripartite-Swiss-Roll.** (a-c) PCA was applied to three subsampled versions of the Tripartite-Swiss-roll, each corresponding to the combination of two groups: a) red vs blue groups; b) blue vs green groups; c) red vs green groups.

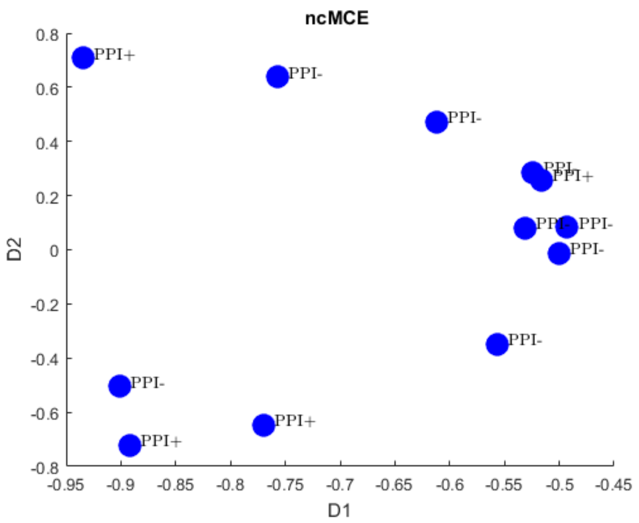


**Supplementary Figure 6. MCE on gastric biopsies dataset (Paroni Sterbini *et al.*), restricted to PPI-treated patients.** The plot shows the MCE result (ncMCE) on the gastric biopsies dataset, restricted to PPI-treated patients. There is no internal separation of the samples related to *H. pylori* infection (PPI+ vs PPI-).

**PSI-ROC**

| Method | Paroni Sterbini | Trust | Amir3 | Trust | Amir4 | Trust |
|---|---|---|---|---|---|---|
| **MCE** | 0.90±0.007 | 0.005±0.001 | 0.91±0.011 | 0.012±0.002 | 0.90±0.007 | 0.013±0.002 |
| **HD** | 0.89±0.004 | 0.004±0.001 | 0.96±0.005 | 0.006±0.002 | 0.98±0.004 | 0.001±0.000 |
| **PCA** | 0.85±0.005 | 0.011±0.001 | 0.88±0.008 | 0.019±0.005 | 0.87±0.007 | 0.021±0.004 |
| **NMDS** | 0.85±0.004 | 0.011±0.001 | 0.86±0.007 | 0.026±0.004 | 0.85±0.007 | 0.028±0.004 |
| **MDSbc** | 0.81±0.004 | 0.028±0.002 | 0.86±0.007 | 0.018±0.003 | 0.86±0.009 | 0.026±0.006 |
| **MDSwUF** | 0.83±0.004 | 0.019±0.002 | 1.00±0.000 | 0.001±0.000 | 0.89±0.008 | 0.016±0.005 |
| **MDStyc** | 0.84±0.005 | 0.015±0.002 | 0.87±0.007 | 0.016±0.002 | 0.86±0.010 | 0.022±0.005 |

**PSI-PR**

| Method | Paroni Sterbini | Trust | Amir3 | Trust | Amir4 | Trust |
|---|---|---|---|---|---|---|
| **MCE** | 0.95±0.004 | 0.003±0.000 | 0.93±0.009 | 0.011±0.002 | 0.90±0.009 | 0.013±0.004 |
| **HD** | 0.94±0.002 | 0.002±0.000 | 0.96±ß.005 | 0.004±0.001 | 0.99±0.004 | 0.001±0.000 |
| **PCA** | 0.90±0.004 | 0.006±0.001 | 0.90±0.008 | 0.013±0.003 | 0.89±0.007 | 0.014±0.004 |
| **NMDS** | 0.90±0.003 | 0.006±0.001 | 0.88±0.008 | 0.018±0.003 | 0.88±0.007 | 0.013±0.003 |
| **MDSbc** | 0.85±0.005 | 0.024±0.003 | 0.89±0.007 | 0.009±0.002 | 0.89±0.008 | 0.010±0.002 |
| **MDSwUF** | 0.87±0.005 | 0.014±0.002 | 1.00±0.000 | 0.001±0.000 | 0.91±0.007 | 0.014±0.004 |
| **MDStyc** | 0.90±0.004 | 0.007±0.001 | 0.89±0.007 | 0.009±0.002 | 0.89±0.008 | 0.012±0.003 |

**Supplementary Table 2. Average PSI-ROC and PSI-PR best results and trustworthiness with standard error on the real datasets, when applying Leave-one-out-cross-validation (LOOCV).** The table shows the average best results of PSI for sample separation in the space of the first two dimensions of embedding based on the well-known metrics Area Under the ROC-Curve (PSI-ROC) and Area Under the Precision-Recall curve (PSI-PR) (regardless of the normalization and type of correlation, and the type of MCE) performed in each of the three different datasets presented in the article (Paroni Sterbini, Amir3 and Amir4), and are represented as mean values +/- the respective standard error. This was done by applying Leave-one-out-cross-validation (LOOCV) where for each dataset the average results and the respective standard error are obtained from the best results of each leave-one-out cross-validation of the dataset (where one sample per time was removed from the dataset and then put back after the analysis was done).

**PSI-ROC**

| Method | Paroni Sterbini | Trust | Amir3 | Trust | Amir4 | Trust | mean |
|---|---|---|---|---|---|---|---|
| **HD** | 0.93 | 0.000999 | 0.92 | 0.000999 | 0.94 | 0.000999 | 0.93 |
| **MDSwUF** | 0.84 | 0.008991 | 0.98 | 0.000999 | 0.88 | 0.000999 | 0.90 |
| **MCE** | 0.83 | 0.006993 | 0.92 | 0.008991 | 0.91 | 0.000999 | 0.89 |
| **PCA** | 0.92 | 0.000999 | 0.89 | 0.008991 | 0.83 | 0.008991 | 0.88 |
| **MDStyc** | 0.83 | 0.014319 | 0.89 | 0.016983 | 0.84 | 0.016983 | 0.86 |
| **NMDS** | 0.86 | 0.007659 | 0.88 | 0.016983 | 0.81 | 0.032967 | 0.85 |
| **MDSbc** | 0.81 | 0.024642 | 0.88 | 0.016983 | 0.84 | 0.008991 | 0.84 |

**PSI-PR**

| Method | Paroni Sterbini | Trust | Amir3 | Trust | Amir4 | Trust | mean |
|---|---|---|---|---|---|---|---|
| **HD** | 0.97 | 0.000999 | 0.92 | 0.000999 | 0.95 | 0.000999 | 0.95 |
| **MCE** | 0.93 | 0.004662 | 0.94 | 0.008991 | 0.92 | 0.000999 | 0.93 |
| **MDSwUF** | 0.88 | 0.003663 | 0.99 | 0.000999 | 0.90 | 0.000999 | 0.92 |
| **PCA** | 0.96 | 0.000999 | 0.89 | 0.000999 | 0.86 | 0.000999 | 0.91 |
| **NMDS** | 0.93 | 0.003663 | 0.90 | 0.015984 | 0.86 | 0.008991 | 0.90 |
| **MDStyc** | 0.91 | 0.004329 | 0.90 | 0.007992 | 0.87 | 0.000999 | 0.89 |
| **MDSbc** | 0.90 | 0.010656 | 0.90 | 0.015984 | 0.88 | 0.000999 | 0.89 |

*Note: all PSI-ROC and PSI-PR can be found in Supplementary Data 9*

**Supplementary Table 3. PSI-ROC and PSI-PR results on the datasets after approximation to the negative binomial distribution.** The table shows the best results of PSI for sample separation in the space of the first two dimensions of embedding, based on the well-known metrics Area Under the ROC-Curve (PSI-ROC) and Area Under the Precision-Recall curve (PSI-PR) (regardless of the normalization and type of correlation, and the type of MCE) performed in each of the three different datasets presented in the article (Paroni Sterbini, Amir3 and Amir4), after approximation to the negative binomial distribution, and the mean performance across all the datasets. Trust denotes the trustworthiness computed for each PSI value. Results are ordered from the best (top) to the worst (bottom) method. For Paroni Sterbini dataset, we show the results for three different labels (PPI-treated, untreated H+ and untreated H-). Instead, for Amir datasets, the PSI values were computed for two groups, i.e. presence or absence of PPI treatment.

**PSI-ROC**

| Method | Paroni Sterbini | Amir3 | Amir4 | mean |
|---|---|---|---|---|
| **HD** | 1 | 2 | 1 | 1.333333 |
| **MDSwUF** | 4 | 1 | 3 | 2.666667 |
| **MCE** | 5 | 2 | 2 | 3 |
| **PCA** | 2 | 4 | 6 | 4 |
| **MDStyc** | 5 | 4 | 4 | 4.333333 |
| **NMDS** | 3 | 6 | 7 | 5.333333 |
| **MDSbc** | 7 | 6 | 4 | 5.666667 |

| PSI-PR | | | | |
|---|---|---|---|---|
| **Method** | **Paroni Sterbini** | **Amir3** | **Amir4** | **mean** |
| **HD** | 1 | 3 | 1 | 1.666667 |
| **MCE** | 3 | 2 | 2 | 2.333333 |
| **MDSwUF** | 7 | 1 | 3 | 3.666667 |
| **NMDS** | 3 | 4 | 6 | 4.333333 |
| **MDStyc** | 5 | 4 | 5 | 4.666667 |
| **MDSbc** | 6 | 4 | 4 | 4.666667 |
| **PCA** | 2 | 7 | 6 | 5 |

**Supplementary Table 4. Rank performance on the datasets after approximation to the negative binomial distribution.** The table shows the rank performance of each method for each in index for sample separation in the space of the first two dimensions of embedding, based PSI-ROC or PSI-PR, for the three datasets presented in the article (Paroni Sterbini, Amir3 and Amir4), after approximation to the negative binomial distribution. Each rank is related with the results obtained in Supplementary Table 3. The results are ordered by the mean performance (fourth column) from the best (top) to the worst (bottom) method.

| **Accuracy** | **Paroni Sterbini et al. (gastric biopsies)** | **Amir3 et al. (esophageal biopsies)** | **Amir4 et al. (gastric fluid)** | **Mean performance** |
|---|---|---|---|---|
| **MC-MCL** | 0.54 (0.58) | 0.75 | 0.63 | 0.64 |
| **MCL** | 0.58 (0) | 0.69 | 0.75 | 0.67 |

*Note: all values can be found in Supplementary Data 10*

**Supplementary Table 5. Clustering results on the datasets after approximation to the negative binomial distribution.** The table shows the best results of clustering (highest accuracies, regardless of the normalization and type of correlation) by Markov Clustering (MCL) and Minimum Curvilinear Markov Clustering, with square rooting the distances, in each of the three different datasets presented in the article

(Paroni Sterbini, Amir3 and Amir4 datasets), after approximation to the negative binomial distribution, and the mean performance (mean of the highest accuracies) across all the datasets.

For Paroni Sterbini dataset, we show the results for three clusters (PPI-treated, untreated H+ and untreated H-) and in brackets the results for four clusters (PPI-treated P&H+, PPI-treated P&H-, untreated H+ and untreated H-). Instead for Amir datasets, the accuracies were computed for two groups, related to presence or absence of PPI treatment.

**PSI-ROC**

| Method | Paroni Sterbini | Trust | Amir3 | Trust | Amir4 | Trust | mean |
|---|---|---|---|---|---|---|---|
| **HD** | 0.93 | 0.000999 | 0.92 | 0.000999 | 0.91 | 0.000999 | 0.92 |
| **MDSwUF** | 0.85 | 0.008991 | 1.00 | 0.000999 | 0.89 | 0.016983 | 0.91 |
| **MDStyc** | 0.86 | 0.003663 | 0.88 | 0.000999 | 0.86 | 0.000999 | 0.86 |
| **PCA** | 0.88 | 0.003663 | 0.86 | 0.024975 | 0.84 | 0.016983 | 0.86 |
| **NMDS** | 0.86 | 0.000999 | 0.86 | 0.024975 | 0.84 | 0.016983 | 0.86 |
| **MCE** | 0.79 | 0.030303 | 0.84 | 0.012987 | 0.92 | 0.000999 | 0.85 |
| **MDSbc** | 0.84 | 0.007326 | 0.88 | 0.000999 | 0.84 | 0.000999 | 0.85 |

**PSI-PR**

| Method | Paroni Sterbini | Trust | Amir3 | Trust | Amir4 | Trust | mean |
|---|---|---|---|---|---|---|---|
| **HD** | 0.97 | 0.000999 | 0.92 | 0.000999 | 0.92 | 0.000999 | 0.94 |
| **MDSwUF** | 0.88 | 0.003663 | 1.00 | 0.000999 | 0.91 | 0.007992 | 0.93 |
| **MDStyc** | 0.93 | 0.000999 | 0.89 | 0.000999 | 0.88 | 0.000999 | 0.90 |
| **PCA** | 0.95 | 0.000999 | 0.85 | 0.03996 | 0.86 | 0.016983 | 0.89 |
| **MDSbc** | 0.88 | 0.009324 | 0.89 | 0.000999 | 0.87 | 0.000999 | 0.88 |
| **NMDS** | 0.91 | 0.000999 | 0.85 | 0.03996 | 0.86 | 0.008991 | 0.87 |
| **MCE** | 0.83 | 0.043623 | 0.86 | 0.008991 | 0.91 | 0.000999 | 0.87 |

*Note: all PSI-ROC and PSI-PR can be found in Supplementary Data 11*

**Supplementary Table 6. PSI-ROC and PSI-PR results on the rarefied datasets.** The table shows the best results of PSI for sample separation in the space of the first two dimensions of embedding, based on the well-known metrics Area Under the ROC-Curve (PSI-ROC) and Area Under the Precision-Recall curve (PSI-PR) (regardless of the normalization and type of correlation, and the type of MCE) performed in each of the three different datasets presented in the article (Paroni Sterbini, Amir3 and Amir4), after being rarefied, and the mean performance across all the datasets. Trust denotes the trustworthiness computed for each PSI value.

Results are ordered from the best (top) to the worst (bottom) method. For Paroni Sterbini dataset, we show the results for three different labels (PPI-treated, untreated H+ and untreated H-). Instead, for Amir datasets, the PSI values were computed for two groups, i.e. presence or absence of PPI treatment.

**PSI-ROC**

| Method | Paroni Sterbini | Amir3 | Amir4 | mean |
|---|---|---|---|---|
| **HD** | 1 | 2 | 2 | 1.666667 |
| **MDSwUF** | 5 | 1 | 3 | 3 |
| **MDStyc** | 3 | 3 | 4 | 3.333333 |
| **PCA** | 2 | 5 | 5 | 4 |
| **NMDS** | 3 | 5 | 5 | 4.333333 |
| **MDSbc** | 6 | 3 | 5 | 4.666667 |
| **MCE** | 7 | 7 | 1 | 5 |

**PSI-PR**

| Method | Paroni Sterbini | Amir3 | Amir4 | mean |
|---|---|---|---|---|
| **HD** | 1 | 2 | 1 | 1.333333 |
| **MDSwUF** | 5 | 1 | 2 | 2.666667 |
| **MDStyc** | 3 | 3 | 4 | 3.333333 |
| **MDSbc** | 5 | 3 | 5 | 4.333333 |
| **PCA** | 2 | 6 | 6 | 4.666667 |
| **MCE** | 7 | 5 | 2 | 4.666667 |
| **NMDS** | 4 | 6 | 6 | 5.333333 |

**Supplementary Table 7. Rank performance on the rarefied datasets.** The table shows the rank performance of each method for each in index for sample separation in the space of the first two dimensions of embedding, based on PSI-ROC or PSI-PR, for the three datasets presented in the article (Paroni Sterbini, Amir3 and Amir4), after being rarefied. Each rank is related with the results obtained in Supplementary Table 6. The results are ordered by the mean performance (fourth column) from the best (top) to the worst (bottom) method.

| Accuracy | Paroni Sterbini et al. (gastric biopsies) | Amir3 et al. (esophageal biopsies) | Amir4 et al. (gastric fluid) | Mean performance |
|---|---|---|---|---|
| **MC-MCL** | 0.58 (0.54) | 0.81 | 0.75 | 0.71 |
| **MCL** | 0.58 (0.42) | 0.69 | 0.75 | 0.67 |

*Note: all values can be found in Supplementary Data 12*

**Supplementary Table 8. Clustering results on the rarefied datasets.** The table shows the best results of clustering (highest accuracies, regardless of the normalization and type of correlation) by Markov Clustering (MCL) and Minimum Curvilinear Markov Clustering, with square rooting the distances, in each of the three different datasets presented in the article (Paroni Sterbini, Amir3 and Amir4 datasets), after being rarefied, and the mean performance (mean of the highest accuracies) across all the datasets.

For Paroni Sterbini dataset, we show the results for three clusters (PPI-treated, untreated H+ and untreated H-) and in brackets the results for four clusters (PPI-treated P&H+, PPI-treated P&H-, untreated H+ and untreated H-). Instead for Amir datasets, the accuracies were computed for two groups, related to presence or absence of PPI treatment.

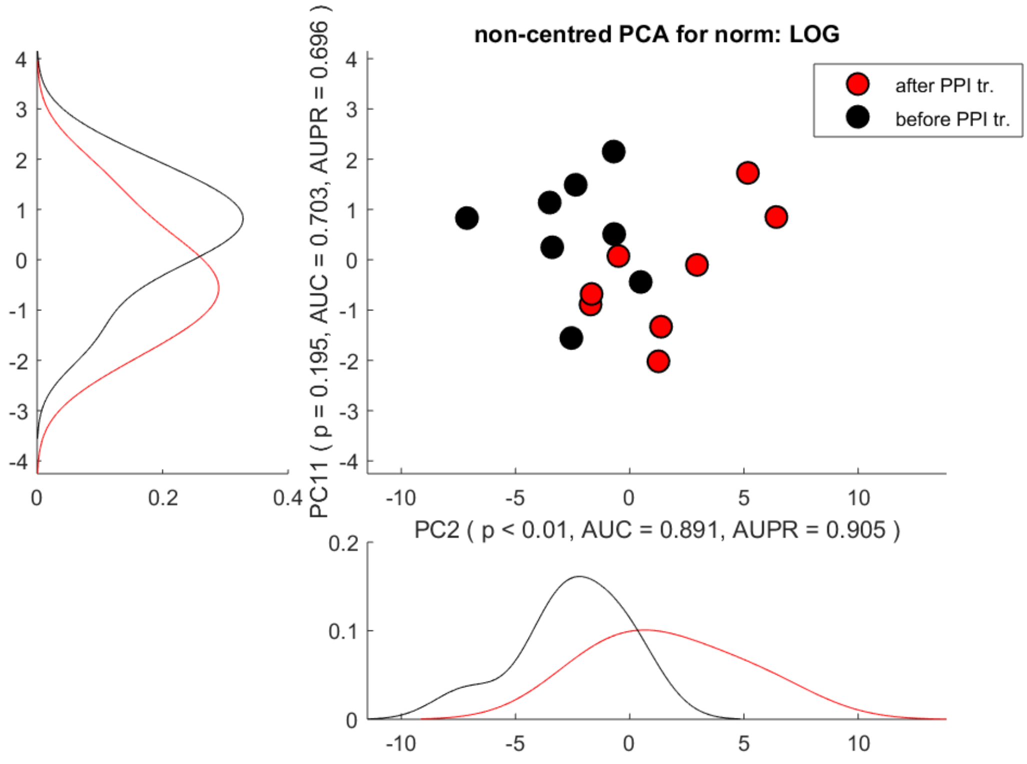


**Supplementary Figure 7. PCA analysis reveals separation related to PPI-treatment in gastric fluid.** Gastric fluid samples before (black dots) and after PPI treatment (red dots) are significantly separated along PC2 (two-sided Mann-Whitney test p-value <0.01).

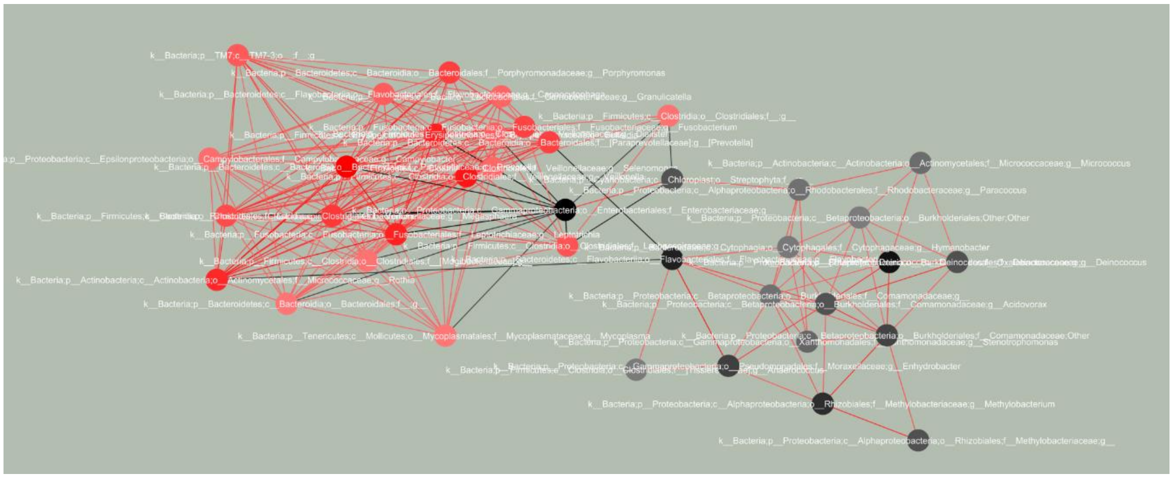

**Supplementary Figure 8. PC-corr network to investigate the effect of PPI treatment on gastric fluid.** The PC-corr network was constructed at cut-off 0.5 according to the loadings of PC2, since PCA could significantly (two-sided Mann-Whitney test $p < 0.01$) separate gastric fluid samples in individuals before and after PPI treatment (Supplementary Figure 7), therefore reflects discriminative network modules related to PPI treatment. Red nodes indicate higher bacterial abundance following PPI treatment (↑after PPI tr.), while black nodes indicate higher bacterial abundance before PPI treatment (↑before PPI tr.).

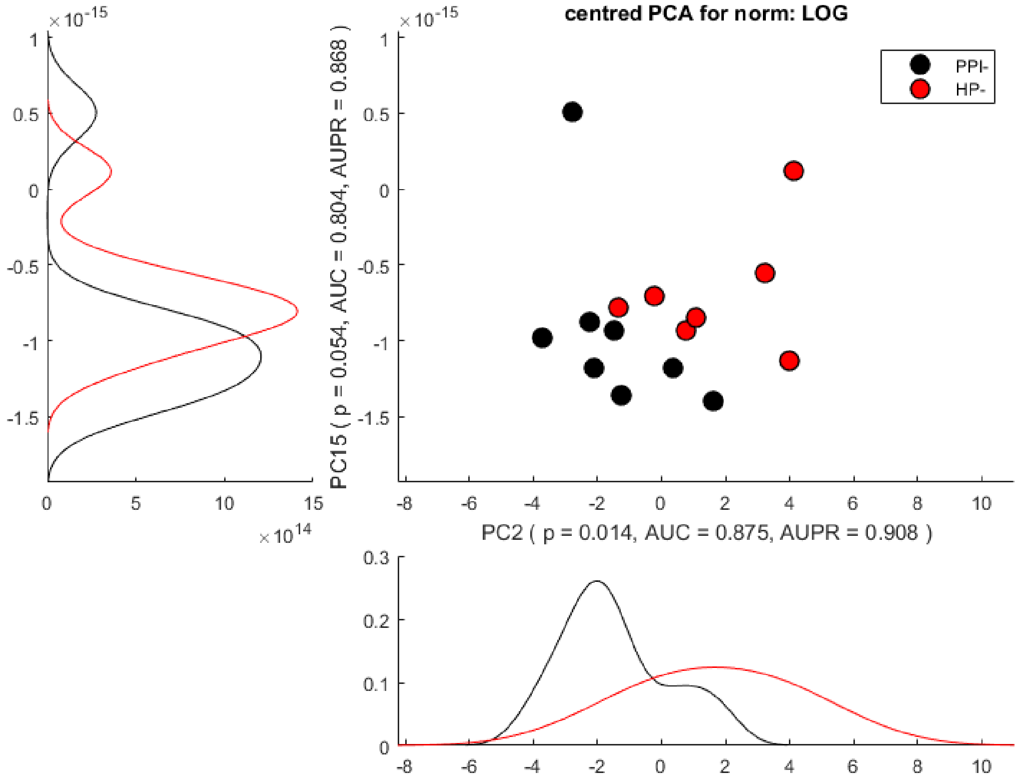


**Supplementary Figure 9. PCA analysis reveals separation related to PPI-treatment in gastric mucosa, in the patients negative to *H. pylori* test.** Linear dimensionality reduction by PCA separates the gastric biopsy samples of PPI-treated *H. pylori*-negative patients (P&H-) (black dots) from the ones of untreated *H. pylori*-negative patients (H-) (red dots) along PC2 and PC15 according to a two-sided Mann-Whitney test (significant p-value along PC2 = 0.014, p-value close to significance along PC15=0.054).

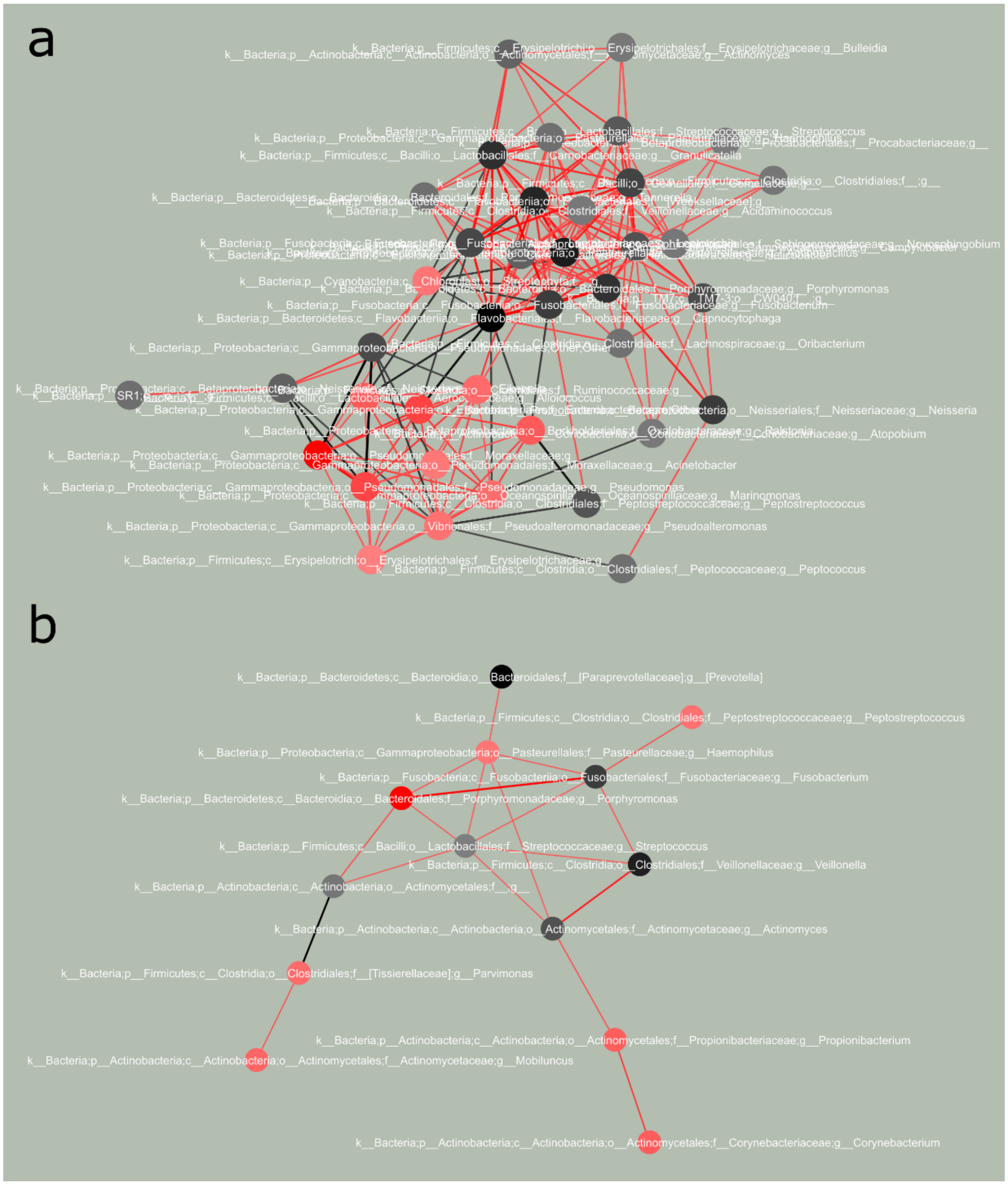


**Supplementary Figure 10. PC-corr network to investigate the effect of PPI treatment on gastric mucosa.** The PC-corr network was constructed at cut-off 0.5 according to the loadings of PC2 (panel a) and PC15 (panel b), since PCA could significantly/closely to significance (according to two-sided Mann-Whitney test p-value along PC2=0.014, p-value along PC15=0.054) separate PPI-treated *H. pylori*-negative patients from *untreated H. pylori*-negative patients (Supplementary Figure 9). Therefore, the discriminative network modules are related to PPI treatment (without *H. pylori* infection). Red nodes indicate higher

bacterial abundance in untreated *H. pylori* negative patients (↑HP-), while black nodes indicate higher bacterial abundance in treated *H. pylori* negative patients (↑PPI-).

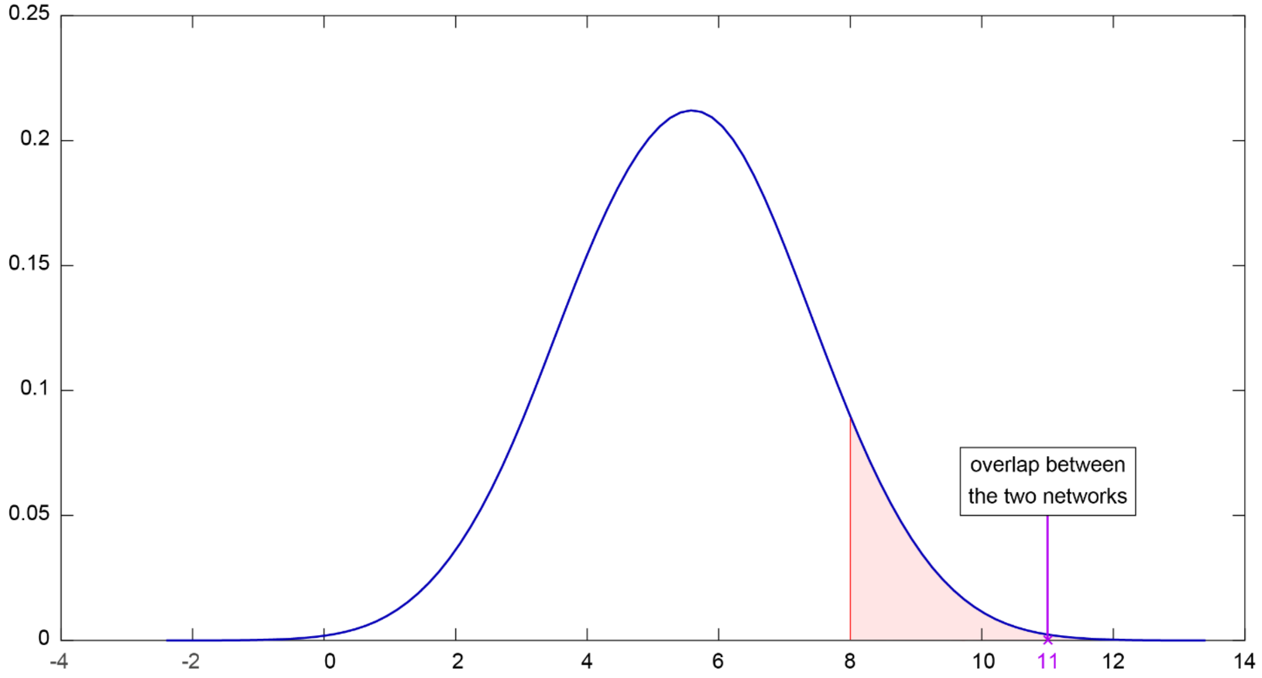


**Supplementary Figure 11. The overlap between Amir *et al.* and Paroni Sterbini *et al.* networks, related to PPI treatment in dyspepsia, is statistically significant and hence it cannot be generated by a random process.** To verify that the overlap between the two networks (violet circle in Figures 3 and 4) is statistically different from a random overlap, we performed a one-sided statistical test based on random resampling, a.k.a. permutation test, of the bacteria in the two networks (repeated 10,000 times). The distribution of the resulting overlap is shown in the above figure, where the red line denotes the 95% percentile hence the tail (red area) on its right sides includes all the overlaps that are significantly different (higher) from random-overlap (p-value<0.05). The overlap between Amir *et al.* and Paroni Sterbini *et al.* networks related to PPI treatment discrimination in dyspepsia (11 bacteria, violet line) is statistically significant (p-value=0.0001), because getting at random this intersection is rare.

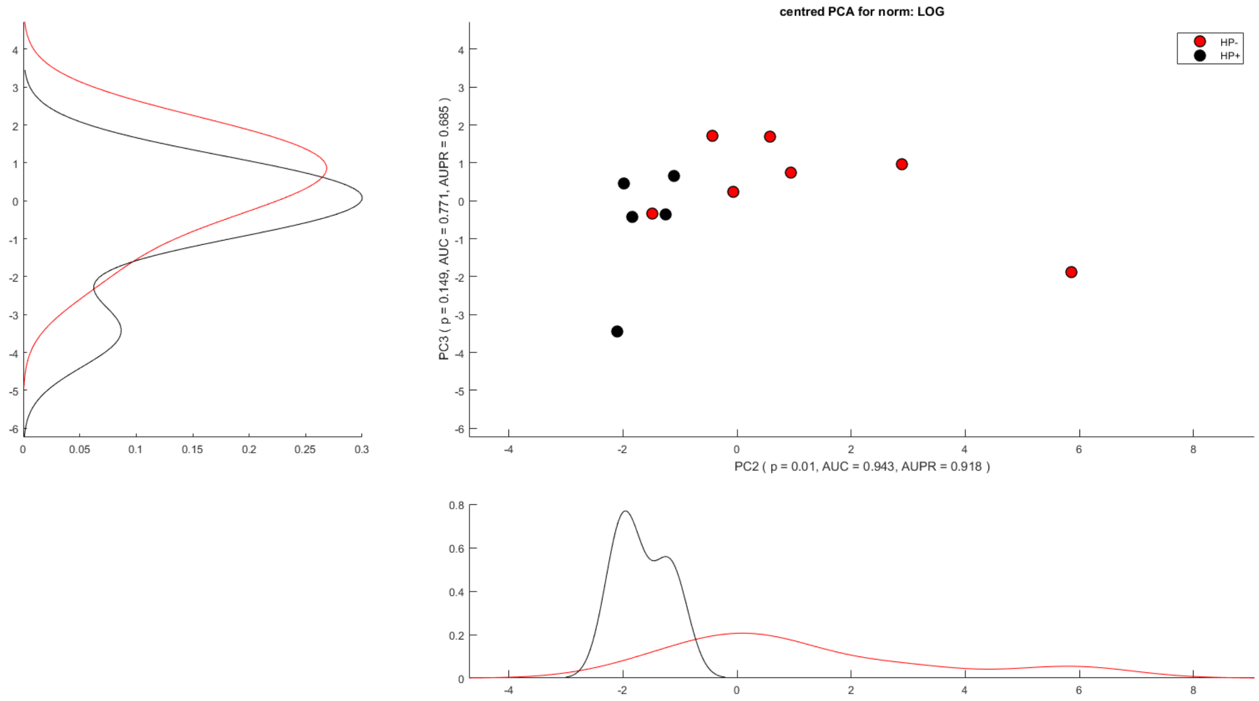


**Supplementary Figure 12. In Paroni Sterbini *et al.* dataset, PCA analysis reveals separation related to *H. pylori* infection in gastric tissue, in the PPI-untreated patients.** Gastric mucosal biopsy sample from *H. pylori*-positive (H+) (black dots) and *H. pylori*-negative (H-) (red dots) PPI-untreated patients were seen to separate along the second principal component (PC2) (two-sided Mann-Whitney test p-value=0.01).

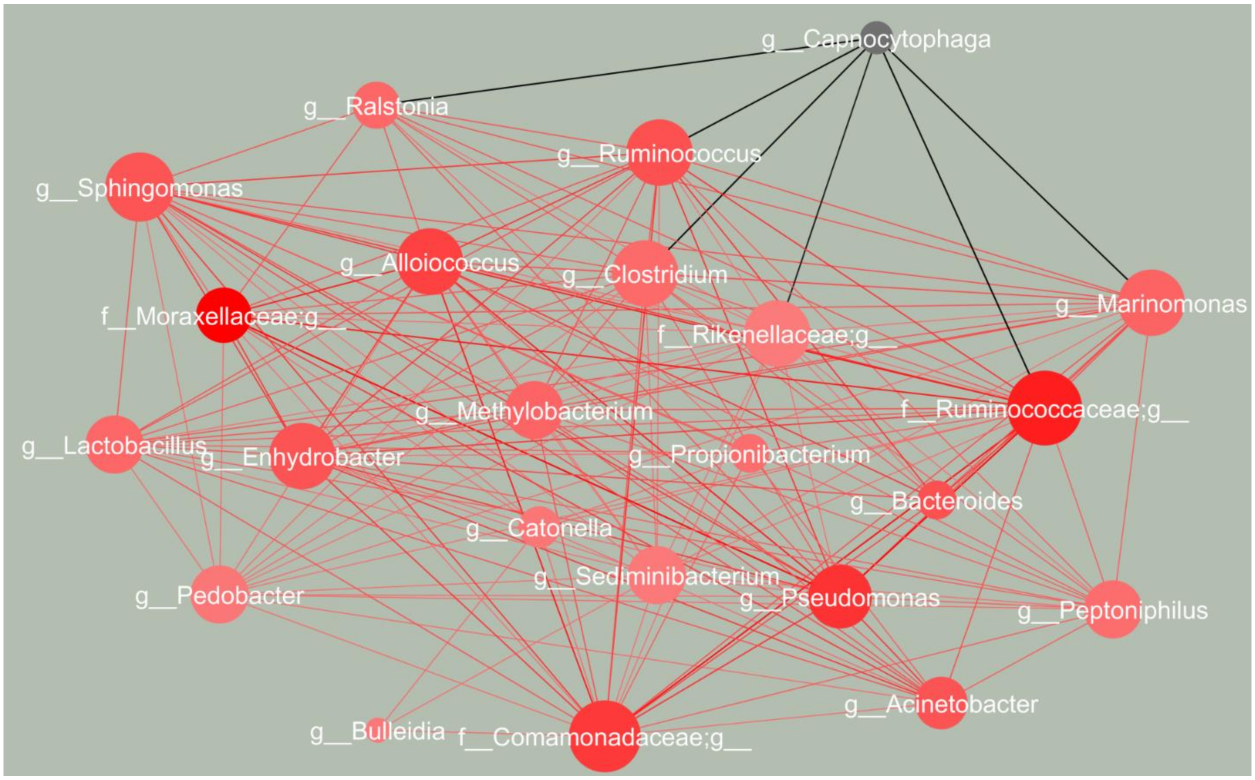


**Supplementary Figure 13. PC-corr network to investigate the effect of *H. pylori* infection on gastric mucosa in Paroni Sterbini *et al.* data.** The PC-corr network was constructed at cut-off 0.5 according to the loadings of PC2, since PCA could significantly (according to two-sided Mann-Whitney test p-value=0.01) separate PPI-untreated *H. pylori*-negative patients from PPI-untreated *H. pylori*-positive patients (Supplementary Figure 12), therefore reflects discriminative network modules related to *H. pylori* infection in gastric mucosa. Red nodes indicate higher bacterial abundance in untreated *H. pylori*-negative patients (↑H-), while black nodes indicate bacterial abundance in untreated *H. pylori*-positive patients (↑H+).

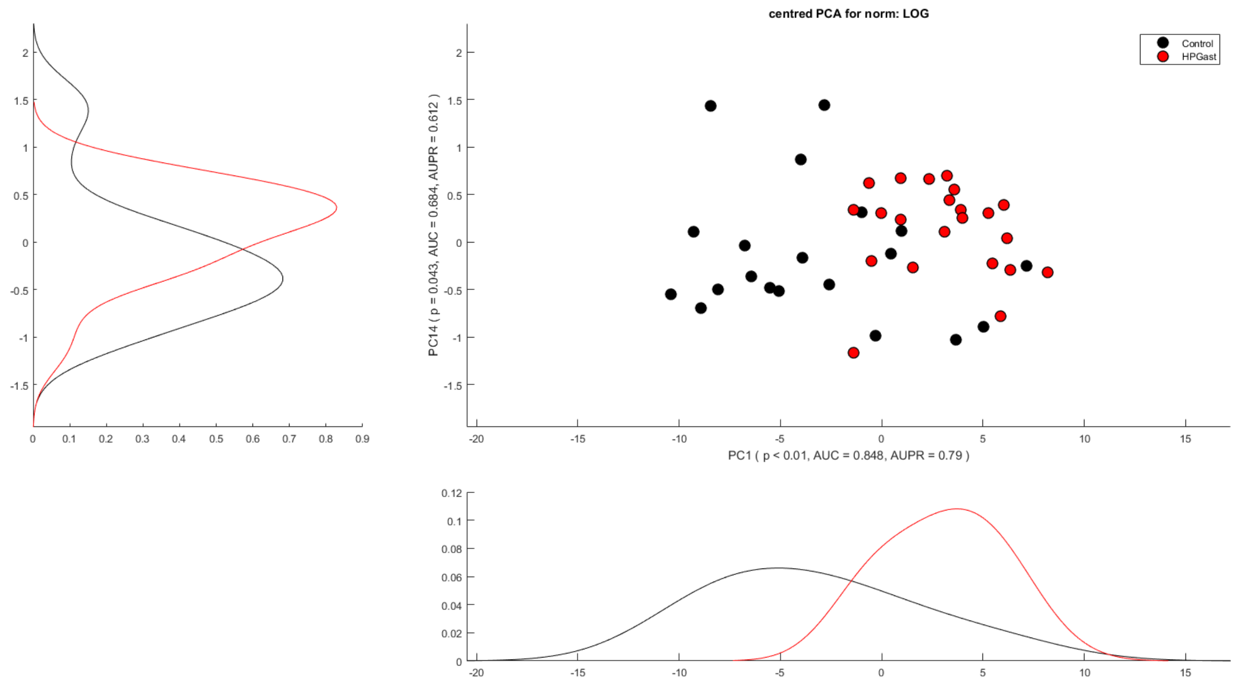


**Supplementary Figure 14. In Parsons *et al. dataset,* PCA analysis can significantly discriminate gastric mucosal biopsy specimens according to *H. pylori*-positivity.** Gastric mucosal biopsy sample from normal stomach group with no evidence of *H. pylori* infection and PPI-untreated (Control, black dots) and *H. pylori* gastritis group positive to *H. pylori* infection and not using PPIs (HPGas, red dots) were seen to separate along the first principal component (PC1) (two-sided Mann-Whitney test p-value<0.01).

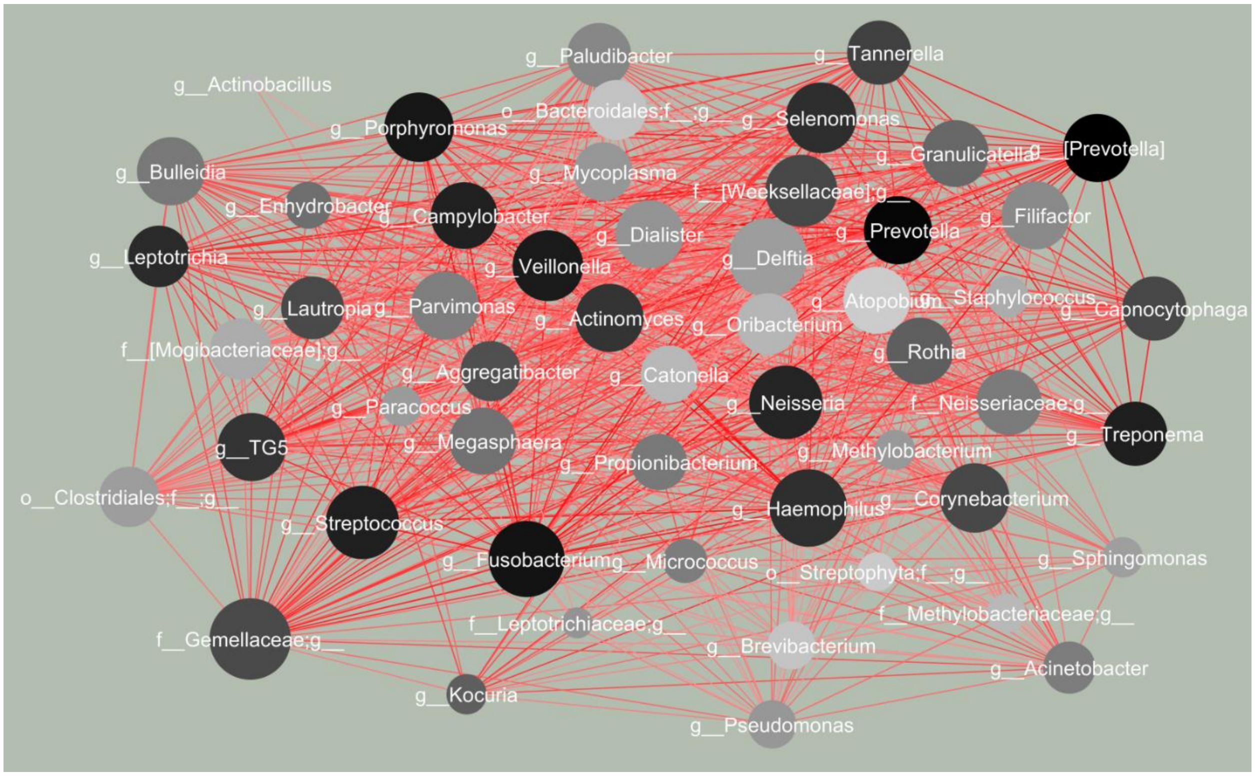


**Supplementary Figure 15. PC-corr network to investigate the effect of *H. pylori* infection on gastric mucosal microbiota in Parsons *et al.* data.** The PC-corr network was constructed at cut-off 0.5 according to the loadings of PC1, since PCA could significantly (according to the two-sided Mann-Whitney test; p-value<0.01) separate patients in the normal stomach group (with no evidence of *H. pylori* infection and PPI-untreated, Control) from patients with *H. pylori* gastritis (positive to *H. pylori* infection and not using PPIs, HPGast) (Supplementary Figure 14), therefore reflects discriminative network modules related to *H. pylori* infection in gastric mucosa. All the bacteria, that are represented by black nodes, have higher abundance in control group (↑Control), that is their abundance is decreased in the presence of *H. pylori* infection (↓HPGast).

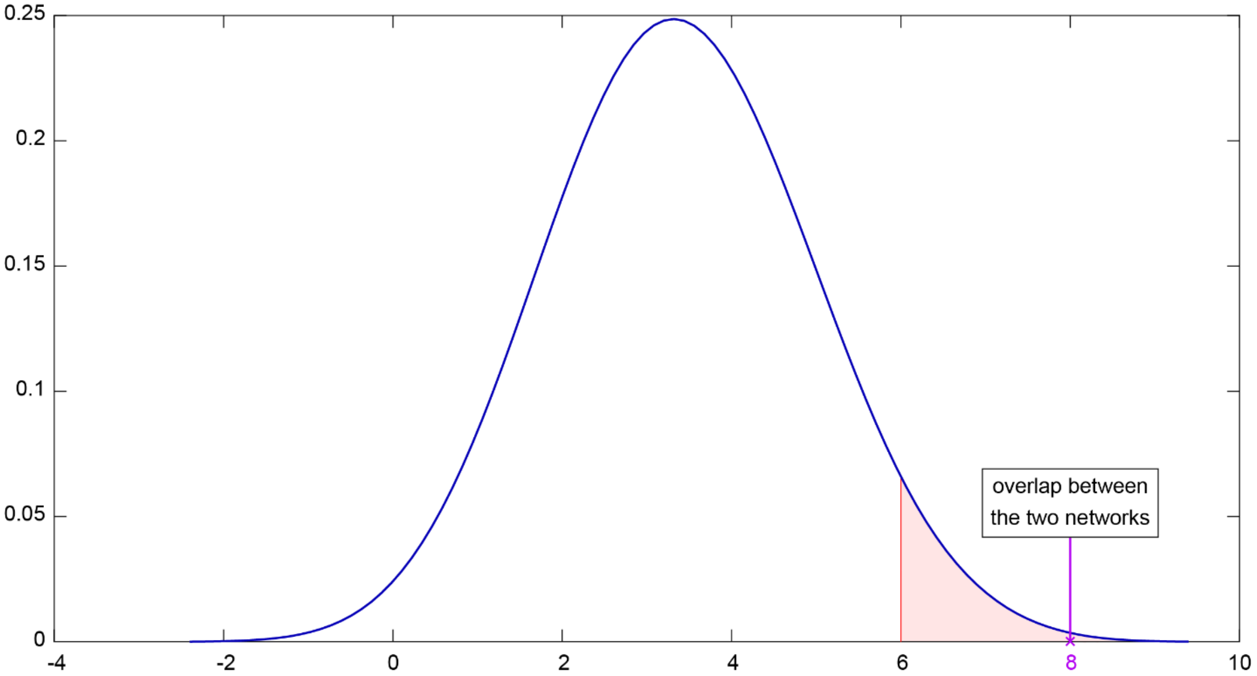


**Supplementary Figure 16. The overlap between Paroni Sterbini *et al.* and Parsons *et al.* networks, exemplifying the effect of *H. pylori* infection on gastric mucosal microbiota, is statistically different from a random overlap.** To verify that the overlap between the two networks (violet circle in Figure 5) is statistically different from a random overlap, we performed a one-sided statistical test based on random resampling, a.k.a. permutation test, of the bacteria in the two networks (repeated 10,000 times). The found intersection (8 bacteria, violet line) is significantly different (p-value=1.00e-04) from a random resampling overlap considering a level of significance of 0.05 (with corresponding critical region in red and critical value denoted with red line), meaning the probability of obtaining at random the same intersection is very low.

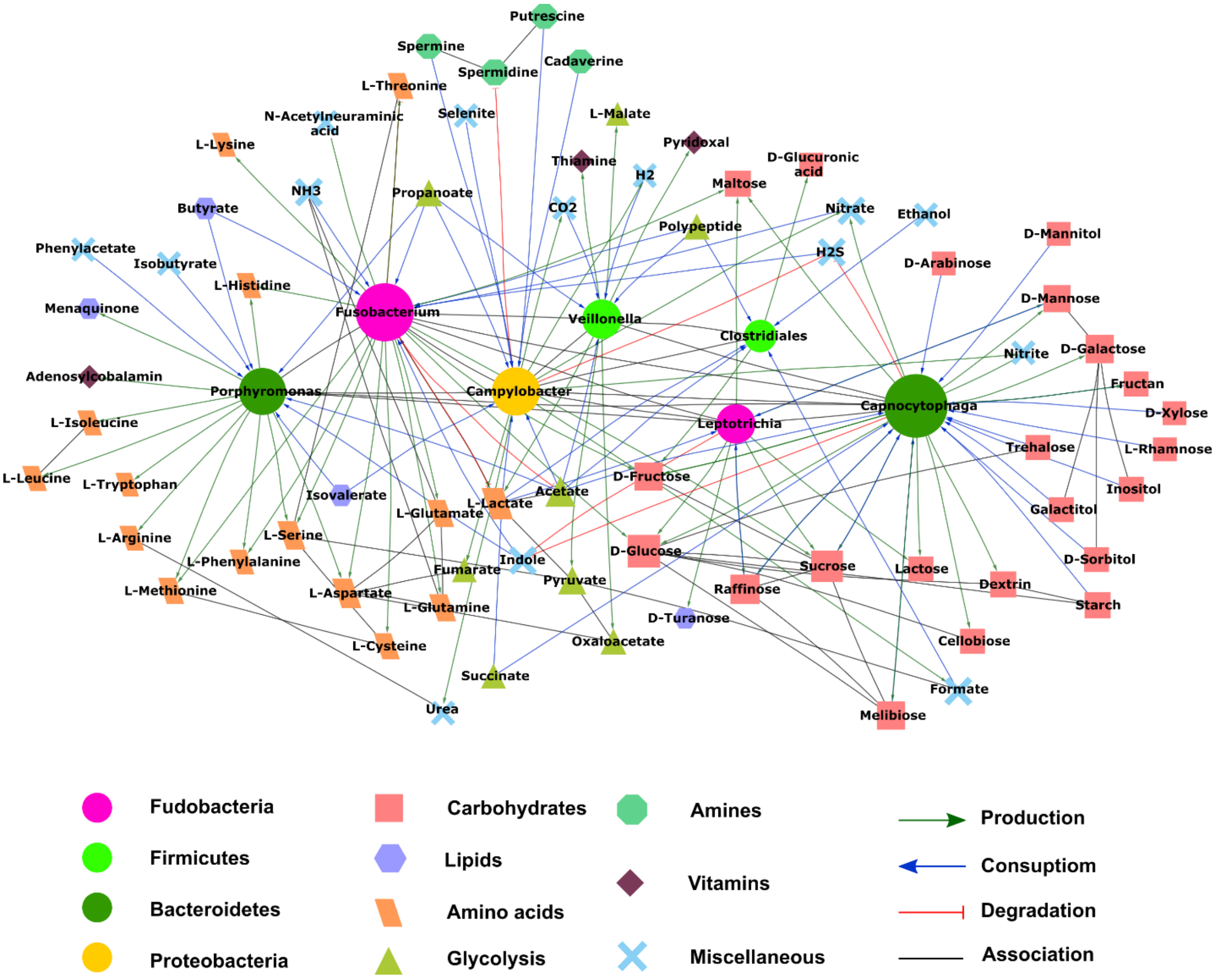


**Supplementary Figure 17. Full PPI-affected bacteria-metabolite network in gastric environment of dyspeptic patients.** Bacteria-metabolite network representation. Bacteria were derived from Figure 4 and represents the consensus network (confirmed in two datasets: gastric mucosa from Paroni Sterbini et al. [2] and gastric fluid from Amir et al. [15]) with PPI-affected bacteria nodes that present information on metabolite interaction in [16]. Consequently, metabolite nodes are the metabolites in [16] interacting with the bacteria network; different node shapes and colours refer to different metabolite classes (carbohydrates, lipids, amino acids, glycolysis, amines, vitamins, miscellaneous), whereas bacteria colours were maintained from Figure 4.

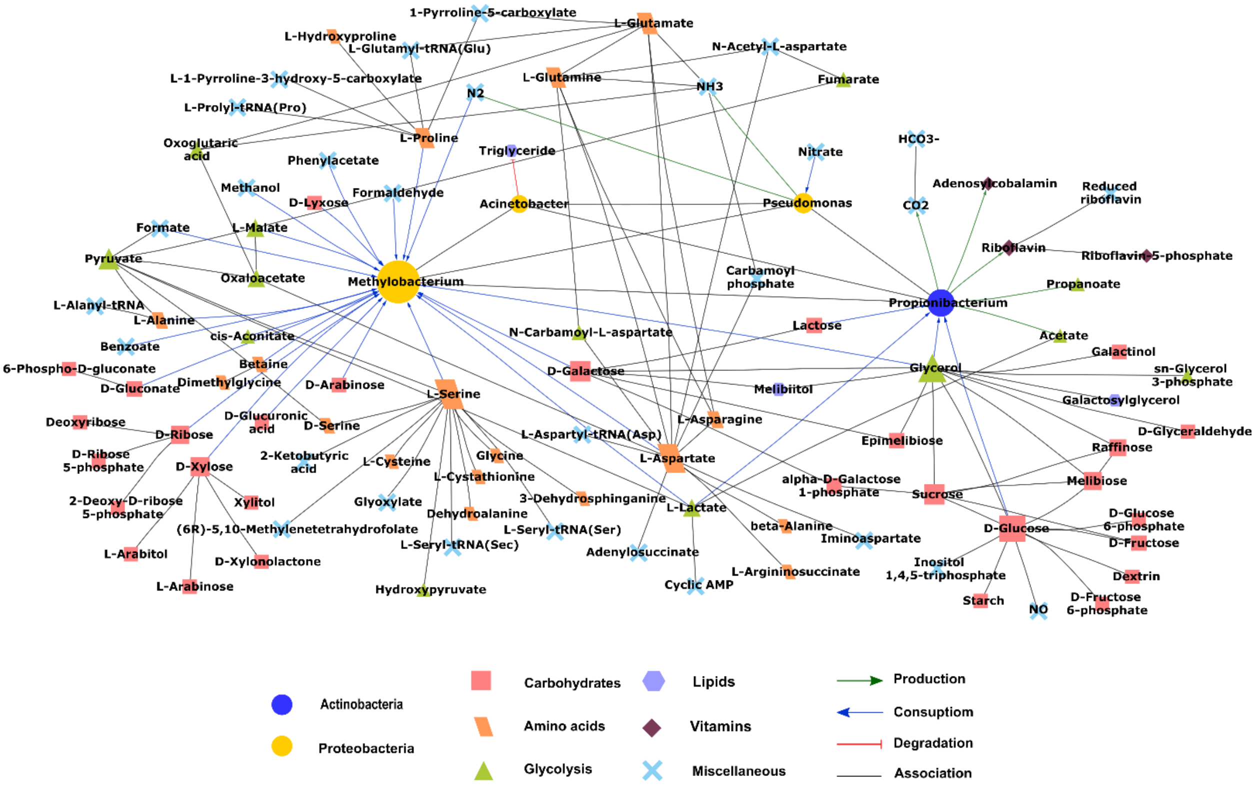


**Supplementary Figure 18. Full *H. Pylori*-affected bacteria-metabolite network in gastric environment of dyspeptic patients.** First neighbor bacteria-metabolite network representation. Bacteria were derived from Figure 5 and represents the consensus network (confirmed in two different datasets of gastric mucosa: Paroni Sterbini et al. [2] and Parsons et al. [17]) with *H. Pylori*-affected bacteria nodes that present information on metabolite interaction in [16]. Consequently, metabolite nodes are metabolites in [16] interacting with the bacteria network, with first neighbour metabolite expansion derived from KEGG pathways; different node shapes and colours refer to different metabolite classes (carbohydrates, lipids, amino acids, glycolysis, vitamins, miscellaneous), whereas bacteria colours were maintained from Figure 5.

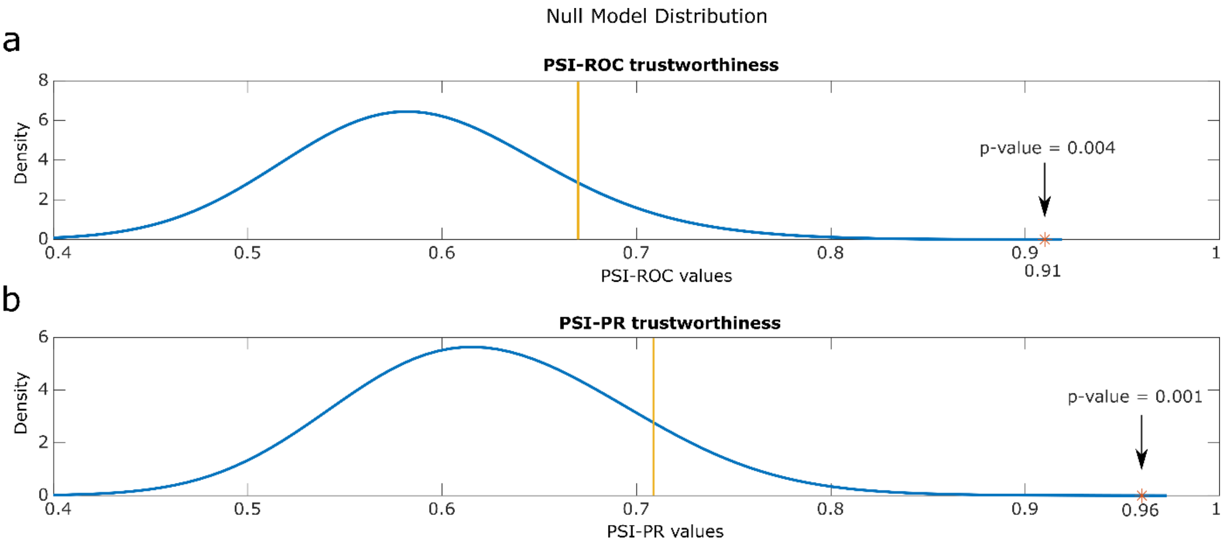


**Supplementary Figure 19. Example of trustworthiness computation.** The figure reports (by employing a one-sided approximated permutation test) the null model distribution obtained for the MCE embedding separation by reshuffling 1000 times the labels of the Paroni Sterbini dataset. (a) Null model distribution for PSI-ROC; (b) null model distribution for PSI-PR. x-axis reports the respective PSI measure values; y-axis reports the density distribution values for the respective PSI measure. The vertical yellow lines denote the 95 percentile of the respective distributions. For the case of MCE embedding, the detected PSI-ROC and PSI-PR values (pointed by the arrows on the right tale of the distribution) are clearly significant (<0.05).

| Dataset | Primer's name | Sequence |
|---|---|---|
| Paroni Sterbini [2] | 28F | 5'TTTGATCNTGGCTCAG |
| Paroni Sterbini [2] | 519R | 5'GTNTTACNGCGGCKGCTG |
| Amir 3 and 4 [15] | 939F | 5'TTGACGGGGGCCCGCAC |
| Amir 3 and 4 [15] | 1492R | 5'TACCTTGTTACGACTT |
| Parsons [17] | 27F-modified | 5'ACACTCTTTCCCTACACGACGCTCTTCCGATCTNNNNNAGAGTTTGATCMTGGC TCAG |
| Parsons [17] | 388R-modified | 5'GTGACTGGAGTTCAGACGTGTGCTCTTCCGATCTGCTGCCTCCCGTAGGAGT |

**Supplementary Table 9. List of primers for each dataset.** The table shows the list of primers, names and sequences, used in the obtainment of datasets Paroni Sterbini, Amir3, Amir4 and Parsons.

**Supplementary Data 1. Excel file with class segregation p-value significance at different embedding dimensions.** The table shows the Mann-Whitney p-values and respective trustworthiness for each dimension in the embedding produced by the dimensionality reduction techniques. Each sheet shows the p-value significance at different normalizations. Significant values (< 0.05) are highlighted in red.

**Supplementary Data 2. Excel file with PSI performances on 16S rRNA gene amplicons data.** The table shows the PSI-ROC and PSI-PR performances from the embedding of the different dimensionality reduction techniques including the different normalizations. Each sheet correspond to a different dataset (Paroni Sterbini, Amir 3 and Amir 4) with exception to the last sheet which contains the summary of performances across all datasets.

**Supplementary Data 3. Excel file with Paroni Sterbini data.** The first sheet shows the percentage of abundance of each bacterium for each sample while the second sheet shows the bacterial absolute abundance raw counts for each sample.

**Supplementary Data 4. Excel file with LDA for dimension reduction.** Information on Fold Cross-Validations and respective error for supervised linear dimensionality reduction by LDA.

**Supplementary Data 5. Excel file with clustering results on real data.** Accuracy performance for the clustering approaches on real datasets for different normalizations and data pre-processing. First sheet: Paroni Sterbini data with 3 clusters. Second sheet: Paroni Sterbini data with 4 clusters. Third sheet: Amir 3 data. Fourth sheet: Amir 4 data.

**Supplementary Data 6. CSV file with PPI-affected bacteria-metabolite network pathway enrichment analysis.** Full list of KEGG pathways enriched for the metabolites present in Figure 6 on the PPI-affected

bacteria-metabolite network. The metabolites present in the network are specified for the significant enriched pathways (<0.05) after Benjamini (FDR) correction.

**Supplementary Data 7. CSV file with *H. pylori*-affected bacteria-metabolite network pathway enrichment analysis.** Full list of KEGG pathways enriched for the metabolites present in Figure 7 on the *H. pylori*-affected bacteria-metabolite network. The metabolites present in the network are specified for the significant enriched pathways (<0.05) after Benjamini (FDR) correction.

**Supplementary Data 8. Excel file with full results of unsupervised analysis on the 'microbial-like' synthetic dataset.** Full performance results of unsupervised dimension reduction techniques according to PSI-ROC and PSI-PR on the 'microbial-like' synthetic dataset evaluated in the space of the first two dimensions of embedding. HD (high dimension) indicates the separability in the high dimensional space (no dimension reduction) and it represents a reference to compare with the separability after dimension reduction. For each PSI value, the respective trustworthiness is reported.

**Supplementary Data 9. Excel file with full PSI-ROC and PSI-PR results on the datasets after approximation to the negative binomial distribution.** The file shows the full performance results of PSI for sample separation in the space of the first two dimensions of embedding, based on PSI-ROC and PSI-PR for different normalizations and performed in each of the three different datasets presented in the article (Paroni Sterbini, Amir3 and Amir4), after approximation to the negative binomial distribution with respective trustworthiness computed for each PSI value. For Paroni Sterbini dataset, we show the results for three different labels (PPI-treated, untreated H+ and untreated H-). Instead, for Amir datasets, the PSI values were computed for two groups, i.e. presence or absence of PPI treatment.

**Supplementary Data 10. Excel file with full clustering results on the datasets after approximation to the negative binomial distribution.** The table shows the full accuracy performances by MCL and MC-

MCL, applying different pre-processing steps, in each of the three datasets presented in the article (Paroni Sterbini, Amir3 and Amir4 datasets), after approximation to the negative binomial distribution. For Paroni Sterbini dataset, we show the results for three and four clusters (sheets 1 and 2). Instead for Amir datasets (sheets 3 and 4), the accuracies were computed for two groups, related to presence or absence of PPI treatment.

**Supplementary Data 11. Excel file with full PSI-ROC and PSI-PR results on the rarefied datasets.** The file shows the full performance results of PSI for sample separation in the space of the first two dimensions of embedding, based on PSI-ROC and PSI-PR for different normalizations and performed in each of the three different datasets presented in the article (Paroni Sterbini, Amir3 and Amir4), after after being rarefied, and with respective trustworthiness computed for each PSI value. For Paroni Sterbini dataset, we show the results for three different labels (PPI-treated, untreated H+ and untreated H-). Instead, for Amir datasets, the PSI values were computed for two groups, i.e. presence or absence of PPI treatment.

**Supplementary Data 12. Excel file with full clustering results on the rarefied datasets.** The table shows the full accuracy performances by MCL and MC-MCL, applying different pre-processing steps, in each of the three datasets presented in the article (Paroni Sterbini, Amir3 and Amir4 datasets), after being rarefied. For Paroni Sterbini dataset, we show the results for three and four clusters (sheets 1 and 2). Instead for Amir datasets (sheets 3 and 4), the accuracies were computed for two groups, related to presence or absence of PPI treatment.

**Supplementary Data 13. Excel file with ‘microbial-like’ dataset.** The file contains the abundance table of the ‘microbial-like’ synthetic dataset.

**Supplementary Data 14. Excel file with Tripartite-Swiss-Roll dataset.** The file contains the coordinates for each point and respective label for the synthetic dataset tripartite-swiss-roll.

# Supplementary References


1. Alanis-Lobato, G., Cannistraci, C. V., Eriksson, A., Manica, A. & Ravasi, T. Highlighting nonlinear patterns in population genetics datasets. *Sci. Rep.* **5**, 8140 (2015).

2. Paroni Sterbini, F. *et al.* Effects of Proton Pump Inhibitors on the Gastric Mucosa-Associated Microbiota in Dyspeptic Patients. *Appl. Environ. Microbiol.* **82**, 6633–6644 (2016).

3. Tenenbaum, J. B., de Silva, V. & Langford, J. C. A global geometric framework for nonlinear dimensionality reduction. *Science* **290**, 2319–23 (2000).

4. Kurtz, Z. D. *et al.* Sparse and Compositionally Robust Inference of Microbial Ecological Networks. *PLoS Comput. Biol.* **11**, e1004226 (2015).

5. Lo, C. & Marculescu, R. MetaNN: Accurate classification of host phenotypes from metagenomic data using neural networks. *BMC Bioinformatics* (2019) doi:10.1186/s12859-019-2833-2.

6. Wong, R. G., Wu, J. R. & Gloor, G. B. Expanding the UniFrac toolbox. *PLoS One* **11**, e0161196 (2016).

7. Weiss, S. *et al.* Normalization and microbial differential abundance strategies depend upon data characteristics. *Microbiome* **5**, 27 (2017).

8. Navas-Molina, J. A. *et al.* Advancing our understanding of the human microbiome using QIIME. in *Methods in Enzymology* vol. 531 371–444 (2013).

9. Hughes, J. B. & Hellmann, J. J. The application of rarefaction techniques to molecular inventories of microbial diversity. in *Methods in Enzymology* vol. 397 292–308 (2005).

10. McMurdie, P. J., Holmes, S., Hoffmann, C., Bittinger, K. & Chen, Y. Waste Not, Want Not: Why Rarefying Microbiome Data Is Inadmissible. *PLoS Comput. Biol.* **10**, e1003531 (2014).

11. Lozupone, C. & Knight, R. UniFrac: a new phylogenetic method for comparing microbial communities. *Appl. Environ. Microbiol.* **71**, 8228–35 (2005).

12. Lozupone, C., Lladser, M. E., Knights, D., Stombaugh, J. & Knight, R. UniFrac: An effective distance metric for microbial community comparison. *ISME J.* **5**, 169–172 (2011).

13. Schloss, P. D. *et al.* Introducing mothur: Open-source, platform-independent, community-supported software for describing and comparing microbial communities. *Appl. Environ. Microbiol.* (2009) doi:10.1128/AEM.01541-09.

14. Jones, D. L. The Fathom Toolbox for Matlab: multivariate ecological and oceanographic data analysis. *Coll. Mar. Sci. Univ. South Florida, St. Petersburg, FL, USA* (2014).

15. Amir, I., Konikoff, F. M., Oppenheim, M., Gophna, U. & Half, E. E. Gastric microbiota is

altered in oesophagitis and Barrett's oesophagus and further modified by proton pump inhibitors. *Environ. Microbiol.* **16**, 2905–2914 (2014).

16. Lim, R. *et al.* Large-scale metabolic interaction network of the mouse and human gut microbiota. *Sci. Data* (2020) doi:10.1038/s41597-020-0516-5.

17. Parsons, B. N. *et al.* Comparison of the human gastric microbiota in hypochlorhydric states arising as a result of. *PLOS Pathog.* **13**, 1–19 (2017).
