# Supplementary material for: Nonlinear machine learning pattern recognition and bacteria-metabolite multilayer network analysis of perturbed gastric microbiome
Source: Nat Commun. 2021 Mar 26;12:1926. doi: 10.1038/s41467-021-22135-x (PMC7997970; doi:10.1038/s41467-021-22135-x)
Supplement: Supplementary file 18 — Description of Additional Supplementary Files [file 41467_2021_22135_MOESM18_ESM.pdf]

Title: Supplementary Data 1:
Description: Excel file with class segregation p-value significance at different embedding dimensions.

Title: Supplementary Data 2:
Description: Excel file with PSI performances on 16S rRNA gene amplicons data.

Title: Supplementary Data 3:
Description: Excel file with Paroni Sterbini data.

Title: Supplementary Data 4:
Description: Excel file with LDA for dimension reduction.

Title: Supplementary Data 5:
Description: Excel file with clustering results on real data.

Title: Supplementary Data 6:
Description: CSV file with PPI-affected bacteria-metabolite network pathway enrichment analysis.

Title: Supplementary Data 7:
Description: CSV file with H. pylori-affected bacteria-metabolite network pathway enrichment analysis.

Title: Supplementary Data 8:
Description: Excel file with full results of unsupervised analysis on the 'microbiallike' synthetic dataset.

Title: Supplementary Data 9:
Description: Excel file with full PSI-ROC and PSI-PR results on the datasets after approximation to the negative binomial distribution.

Title: Supplementary Data 10:
Description: Excel file with full clustering results on the datasets after approximation to the negative binomial distribution.

Title: Supplementary Data 11:
Description: Excel file with full PSI-ROC and PSI-PR results on the rarefied datasets.

Title: Supplementary Data 12:
Description: Excel file with full clustering results on the rarefied datasets.

Title: Supplementary Data 13:
Description: Excel file with 'microbial-like' dataset.

Title: Supplementary Data 14:
Description: Excel file with Tripartite-Swiss-Roll dataset.
